# Evaluating the usefulness of alignment filtering methods to reduce the impact of errors on evolutionary inferences

**DOI:** 10.1186/s12862-019-1350-2

**Published:** 2019-01-11

**Authors:** Arnaud Di Franco, Raphaël Poujol, Denis Baurain, Hervé Philippe

**Affiliations:** 1 Station d’Ecologie Théorique et Expérimentale de Moulis, CNRS, Moulis, France; 2 Département de Biochimie, Centre Robert-Cedergren, Université de Montréal, Montréal, Québec Canada; 3 InBioS–PhytoSYSTEMS, Unité de Phylogénomique des Eucaryotes, Université de Liège, Liège, Belgium

**Keywords:** Multiple sequence alignment, Profile hidden Markov models, Low similarity segments, Primary sequence error, Phylogeny, Positive selection

## Abstract

**Background:**

Multiple Sequence Alignments (MSAs) are the starting point of molecular evolutionary analyses. Errors in MSAs generate a non-historical signal that can lead to incorrect inferences. Therefore, numerous efforts have been made to reduce the impact of alignment errors, by improving alignment algorithms and by developing methods to filter out poorly aligned regions. However, MSAs do not only contain alignment errors, but also primary sequence errors. Such errors may originate from sequencing errors, from assembly errors, or from erroneous structural annotations (such as incorrect intron/exon boundaries). Even though their existence is acknowledged, the impact of primary sequence errors on evolutionary inference is poorly characterized.

**Results:**

In a first step to fill this gap, we have developed a program called HmmCleaner, which detects and eliminates these errors from MSAs. It uses profile hidden Markov models (pHMM) to identify sequence segments that poorly fit their MSA and selectively removes them. We assessed its performances using > 700 amino-acid MSAs from prokaryotes and eukaryotes, in which we introduced several types of simulated primary sequence errors. The sensitivity of HmmCleaner towards simulated primary sequence errors was > 95%. In a second step, we compared the impact of segment filtering software (HmmCleaner and PREQUAL) relative to commonly used block-filtering software (BMGE and TrimAI) on evolutionary analyses. Using real data from vertebrates, we observed that segment-filtering methods improve the quality of evolutionary inference more than the currently used block-filtering methods. The formers were especially effective at improving branch length inferences, and at reducing false positive rate during detection of positive selection.

**Conclusions:**

Segment filtering methods such as HmmCleaner accurately detect simulated primary sequence errors. Our results suggest that these errors are more detrimental than alignment errors. However, they also show that stochastic (sampling) error is predominant in single-gene evolutionary inferences. Therefore, we argue that MSA filtering should focus on segment instead of block removal and that more studies are required to find the optimal balance between accuracy improvement and stochastic error increase brought by data removal.

**Electronic supplementary material:**

The online version of this article (10.1186/s12862-019-1350-2) contains supplementary material, which is available to authorized users.

## Background

Evolutionary studies require the identification of homologous characters. Except for highly divergent proteins, the recognition of homologous protein-coding genes is generally straightforward because the availability of many positions provides enough statistical power, at least some protein regions being well conserved (i.e., show a high similarity). In contrast, the identification of homology at the residue level, through multiple sequence alignment (MSA), is more difficult. Limited statistical power and low similarity may generate ambiguously aligned regions (AARs). Due to the high combinatorics of sequence alignment, some parts of AARs are expected to be aligned wrongly more often than correctly. Despite efforts in improving alignment methods [1], errors still affect MSAs and may negatively impact subsequent analyses. During phylogenetic inference, they generate a non-phylogenetic signal, conflicting with the genuine (historical) phylogenetic signal in the data [2, 3]. Their presence also inflates estimates of positive selection [4, 5].

A common approach to reduce the impact of alignment errors is a posteriori filtering of MSAs. The rationale of the block-oriented strategy is that alignment errors are in excess in AARs, the variable regions of the proteins (in particular those with a high rate of insertion/deletion). Several software packages [6–12] were designed to identify AARs based on various criteria, such as the stability of the MSA to the guide tree [12] or the validation of a set of rules dependent on the conservation pattern [6, 8, 9]. AARs are expected to contain non-homologous residues in most sequences, but also genuine homologous residues in the remaining sequences. Removal of AARs is therefore expected to simultaneously decrease non-phylogenetic and phylogenetic signal, but the first more than the second. Some studies suggest that block-filtering software improves evolutionary inference [13–16], whereas other authors find support for the opposite [17, 18].

Another source of noise in MSAs are primary sequence errors. These stem from sequencing errors, assembly errors or, in the case of amino-acid MSAs, structural annotation errors (such as incorrect intron/exon boundaries). Fundamentally different from alignment errors, primary sequence errors (especially those affecting only one or a few sequences) are unlikely to be removed by block-filtering programs, except if they are included within AARs. To properly handle such errors, filtering software should be designed to remove amino-acid segments sequence by sequence, instead of block by block.

Besides, primary sequence errors provide a strong non-historical signal that is more likely to bias evolutionary estimates (e.g., by lengthening the corresponding terminal branches in a phylogeny). Accordingly, a few studies have shown that they can be a source of erroneous signal [3] or even drive alignment errors [5]. Yet, this aspect is generally not taken into account while analyzing MSAs. In fact, nothing is known about the relative importance of primary sequence errors versus alignment errors in evolutionary analysis of real MSAs.

Here, we present HmmCleaner, a program dedicated to the detection and removal of primary sequence errors in multiple alignments of protein sequences. It implements an approach looking for low similarity segments specific to one sequence using a profile hidden Markov model (pHMM) built from the whole alignment with HMMER [19]. In the following sections, we first introduce the HmmCleaner principle. Then we explain the optimization of its parameters, characterize its performance by simulating primary sequence errors and compare it to PREQUAL performance [20], a recently released software package with a similar approach based on pairHMM. Then, we address the effect of filtering software on evolutionary analysis. First, we determine whether the use of HmmCleaner avoids the erroneous detection of positive selection when frameshift errors have been voluntarily introduced. Second, using empirical datasets, we compare the effect of segment- and block-filtering methods on evolutionary inferences (single-gene phylogenetic reconstruction and branch length estimation) as a first insight into the relative impact of alignment errors and primary sequence errors.

## Results and discussion

### Overview of HmmCleaner and parameter optimization

**Fig. 1:**
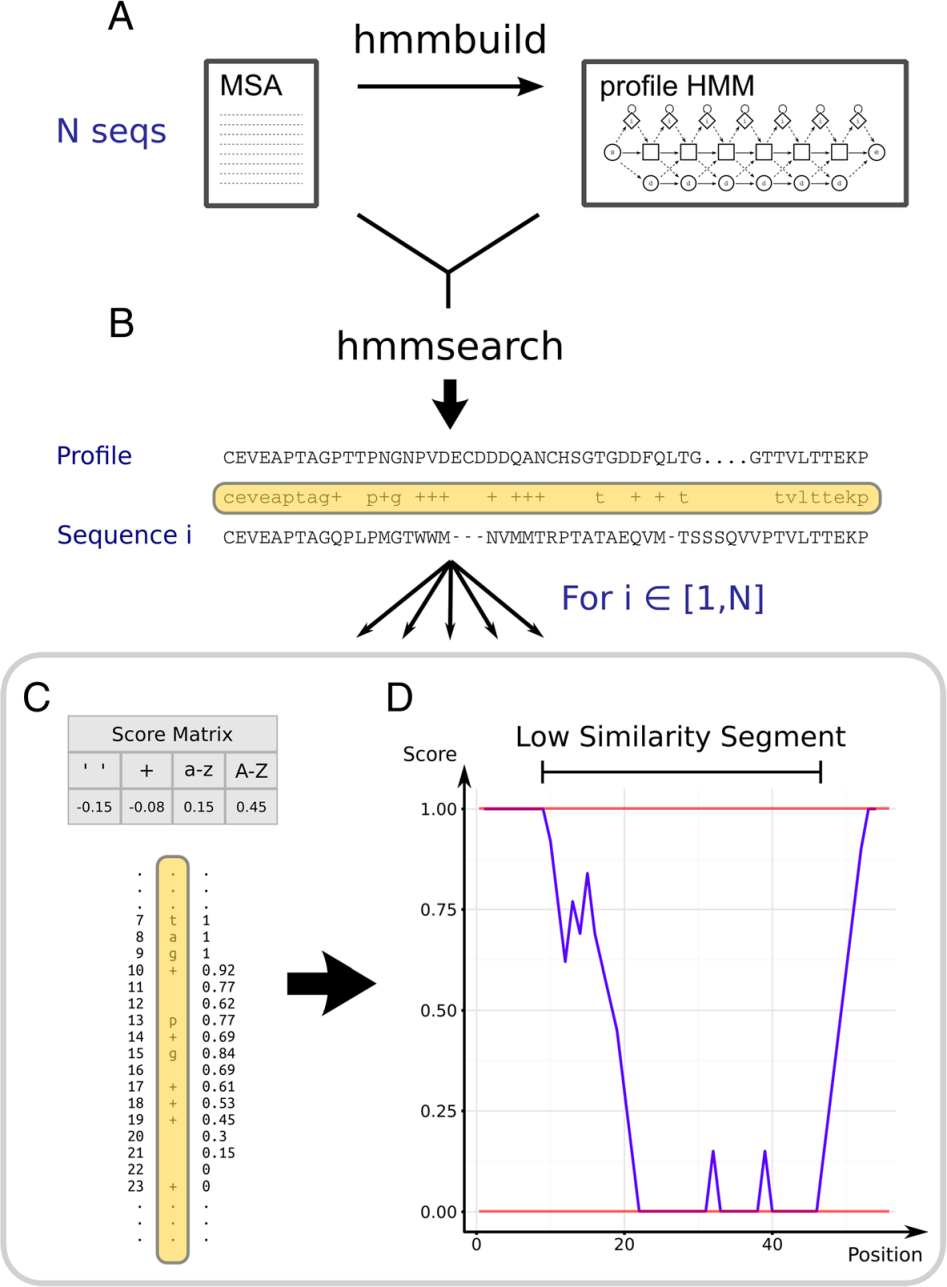
Overview of the four steps of HmmCleaner. **a**. Creation of a pHMM from the MSA. In the diagram representing the pHMM, squares correspond to main states of the model whereas diamonds are insertion states and circles deletion states. **b**. Alignment of one envelope of a given sequence of the MSA. **c**. Computation of the cumulative similarity score based on the analysis of the string of match/mismatch (underlined in yellow) using the four-parameter scoring matrix. **d**. Determination of low similarity segments as continuous sequence stretches with similarity score < 1 in which at least one position reached a null score

HmmCleaner identifies primary sequence errors by detecting low similarity segments in an MSA (Fig. 1). In our framework, low similarity segments are stretches of residues that are highly divergent with respect to the full alignment (in terms of sequence, length or both). They are identified through four steps. First, a pHMM is built from the MSA using HMMER (Fig. 1a); it will be used as the reference, i.e., the underlying model having generated each sequence of the MSA. It can either be built upon (i) all sequences of the MSA (complete strategy) or (ii) all sequences except the currently analyzed one (leave-one-out strategy). Second, each sequence of the MSA is evaluated with the pHMM (Fig. 1b), which yields one profile-sequence alignment per sequence through the heuristic of HMMER [19]. Third, each profile-sequence alignment is analyzed by considering the four categories of match between the sequence and the pHMM consensus using a four-parameters matrix that increases a cumulative similarity score when the residue is expected by the pHMM or decreases it otherwise (Fig. 1c). The evolution of this similarity score depicts the variation of the corresponding sequence fit to the pHMM along its whole length. Fourth, low similarity segments are defined as continuous segments where the similarity score was lower than the maximal value and among which at least one residue reaches a null score (Fig. 1d, see Materials and Methods for details).

To optimize the four parameters of the scoring matrix, we developed a simulator that introduces primary sequence errors into existing MSAs. The principle is to take a genuine protein-coding alignment of nucleotide (nt) sequences and to randomly introduce a unique error in a specified number of sequences. The resulting sequences are then translated into amino acids (aa) before realignment. Here, we chose to generate frameshift errors, each one followed by the opposite (compensatory) mutation after a predefined number of out-of-frame codons. This approach allowed us to use multiple alignments of true protein sequences resulting from real evolutionary processes whereas primary sequence errors are simulated, contrary to Whelan et al. [20], who started from simulated sequences.

**Fig. 2:**
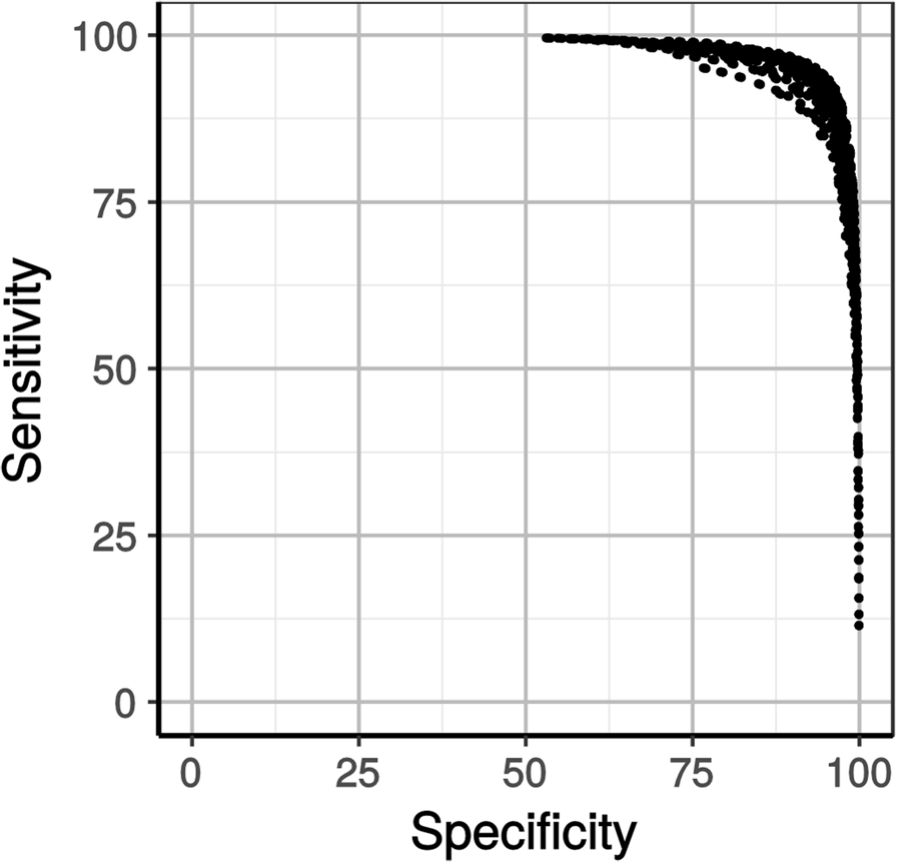
Mean sensitivity and specificity of HmmCleaner towards detection of primary sequence errors introduced in unambiguously aligned regions (UARs). Each dot corresponds to the two means of the values obtained across 80,000 simulations and 3 operational definitions of UARs for one of the 2835 combinations of the 4 parameters of the scoring matrix

Two empirical datasets of 100 MSAs composed of prokaryotic genes (either Euryarchaeota or Cyanobacteria) were submitted to our simulator in four variants of species sampling (5, 10, 25 and 50 species), so as to generate 100 replicates of these 800 combinations containing 1 to 5 primary sequence errors of length 10 to 100 aa. The random sampling of species allowed us to test a large variety of tree shape, given that HMMER implicitly assumes that a star tree topology has generated the alignment. The 80,000 simulated MSAs were then used to explore the effect of the four parameter values on both the sensitivity (detection of truly non-homologous segments) and the specificity (non-detection of genuinely homologous segments) of HmmCleaner. We chose to work only on unambiguously aligned regions (UARs), reasoning that it is more difficult to differentiate non-homologous segments from homologous but highly divergent ones in ambiguously aligned regions (AARs). Indeed, over a grid of 2835 quartets of parameter values (9*c1, 7*c2, 9*c3, 5*c4, see Materials and Methods), HmmCleaner only reached a limited specificity (93%) in AARs (Additional file 1 Figure S1), and this came at the expense of a low sensitivity (22%). In contrast, in UARs (Fig. 2), numerous quartets of parameter values led to bothhigh sensitivity and specificity (> 90%), showing that HmmCleaner reliably detects simulated primary sequence errors. Moreover, these results held true when focusing on either Euryarchaeota or Cyanobacteria, and when varying the operational definition of UARs/AARs (Additional file 1 Figure S2A-D). In contrast, sensitivity was reduced with smaller species samples (Additional file 1 Figure S2E), as expected since pHMMs are more powerful when built from a larger number of sequences.

In the end, we selected as the default matrix the parameter set that maximized the mean sensitivity and the mean specificity across all variations of the conditions of simulation. This set of parameters yields both a global sensitivity and specificity of 94% in UARs (Table 1). It improves specificity compared to the empirically determined parameters present in a previous, unpublished, implementation of HmmCleaner, referred to as version 1.8. However, the usual trade-off between specificity and sensitivity, and the negative effect of smaller numbers of species on sensitivity, led us to define three new additional scoring matrices. First, we created a parameter set to use when the number of sequences in the MSA is large, optimized only from simulations performed with 50 species. This scoring matrix achieved a global sensitivity of 97% and a global specificity of 96% in UARs of species-rich MSAs. Second, for users wishing a low false positive rate, we built a high-specificity matrix reaching a global specificity of 97% while keeping a sensitivity of 88%. Finally, we also generated a matrix that simultaneously addresses these two requirements (see Table 1).

**Table 1:** Sensitivity and specificity of HmmCleaner

matrix name	c1	c2	c3	c4	global sensitivity	global specificity	sensitivity for 50 seqs	specificity for 50 seqs
default	−0.150	−0.080	0.150	0.450	94.26%	94.42%	98.42%	93.97%
species-rich	−0.175	− 0.175	0.150	0.400	85.70%	97.02%	97.36%	96.48%
high-specificity	−0.125	−0.125	0.175	0.400	88.89%	97.34%	96.64%	97.08%
species-rich and high- specificity	−0.125	− 0.125	0.150	0.400	74.03%	98.78%	94.11%	98.56%
HmmCleaner 1.8	−0.300	−0.100	0.200	0.500	94.56%	91.57%	98.74%	90.73%

The four new scoring matrices provided with HmmCleaner v2 and the scoring matrix equivalent to HmmCleaner v1.8 for comparison. c1-c4: values of the elemental scores for the four levels of residue conservation provided by HMMER. Global sensitivity and specificity were computed across all conditions of simulation, whereas the last two columns only used the most species-rich MSAs

Since the default scoring matrix had been optimized using MSAs aligned with MAFFT using the L-INS-i algorithm and by building a single pHMM per MSA (complete strategy), we checked that parameter optimization was robust to a change of the aligner software and the HmmCleaner strategy. While both sensitivity and specificity revealed virtually insensitive to the aligner software (MAFFT [21] with two different algorithms, MUSCLE [22] and Clustal Omega [23], Additional file 1 Figure S3), the leave-one-out strategy showed a slightly higher sensitivity (98% versus 97%), but a lower specificity (79% versus 86%), with respect to the complete strategy (Additional file 1 Figure S4). Given these results and the computational burden implied by the leave-one-out strategy, we decided to stick to the complete strategy in the remaining of this article.

### Impact of error length, number and conservation context on HmmCleaner performance

**Fig. 3:**
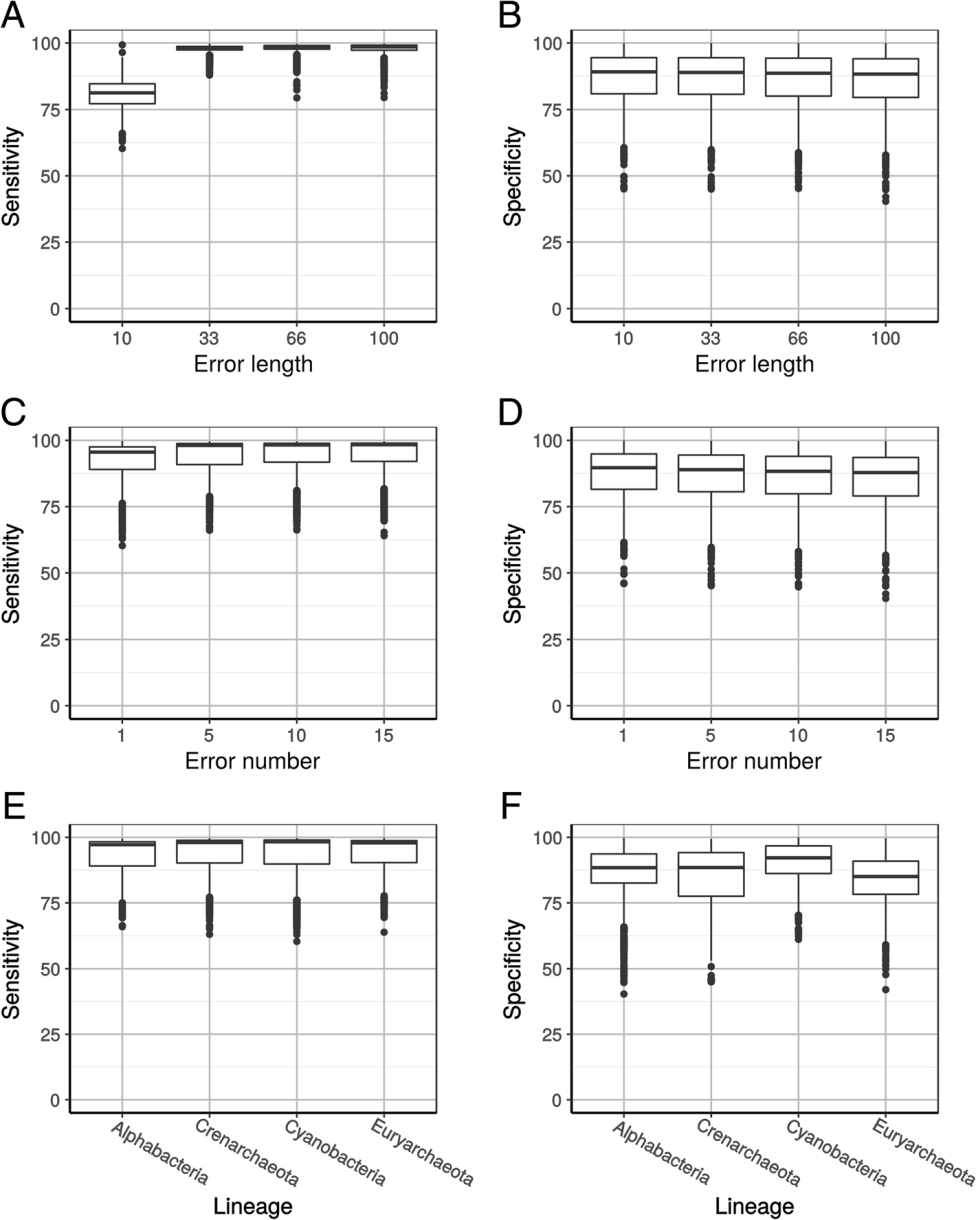
Impact of the length and number of primary sequence errors, and of the prokaryotic lineage, on sensitivity (**a**,**c**,**e**) and specificity (**b**,**d**,**f**) of HmmCleaner used with the default scoring matrix. **a**,**b**. Effect of primary sequence error length. **c**,**d**. Effect of the number of primary sequence errors. **e**,**f**. Effect of the prokaryotic lineage. Box-plots were computed across all considered MSAs and values are means averaged over the different conditions of simulation

To investigate the impact of the length and number of primary sequence errors on the sensitivity and specificity of HmmCleaner, 640,000 simulations introducing a total of 4,960,000 individual errors were run on MSAs from 4 different prokaryotic lineages (Alphaproteobacteria and Crenarchaeota in addition to the Cyanobacteria and Euryarchaeota used so far). Introduced errors were 10 to 100 aa in length and 1 to 15 in number per MSA of 25 randomly selected sequences. Neither error length or number, nor MSA lineage substantially impacted specificity and sensitivity of HmmCleaner, except in two cases (Fig. 3). First, specificity decreased with evolutionary depth and diversity of the clade (Cyanobacteria < Alphaproteobacteria < Crenarchaeota < Euryarchaeota, Fig. 3f), which is in agreement with the idea that HmmCleaner wrongly detects some homologous low similarity segments (see below). Second, sensitivity was severely impacted by short error lengths (Fig. 3a), owing to the limited statistical power provided by such short primary sequence errors.

The same kind of simulations were used to compare HmmCleaner and its different parameter sets to PREQUAL (Additional file 1 Table S1, Additional file 1 Figure S5). Overall, PREQUAL showed higher specificity (92.4% vs 86.7%) and lower sensitivity (83.3% vs 93.3%) compared to the new HmmCleaner default scoring matrix. As expected from the known sensitivity/specificity tradeoff, using the specificity-oriented parameter set reduced the difference in specificity (92.4% vs 91.8%) while keeping sensitivity slightly higher than PREQUAL (83.3% vs 86.1%). Only the “large specificity” parameter set surpassed PREQUAL specificity but at the cost of lower sensitivity. The behavior of HmmCleaner and PREQUAL towards error length and taxonomic diversity is similar. However, PREQUAL sensitivity diminished with increasing error numbers, whereas we observed the opposite for HmmCleaner. Our hypothesis is that more errors increase the probability of having overlapping identical errors. As PREQUAL considers the best posterior probability per residue across a series of closely related sequences, only one identical residue is enough to consider it as correct. In summary, the behavior of HmmCleaner and PREQUAL is similar, which could be due to their common reliance on HMM, and HmmCleaner appears more sensitive yet less specific than PREQUAL.

**Fig. 4:**
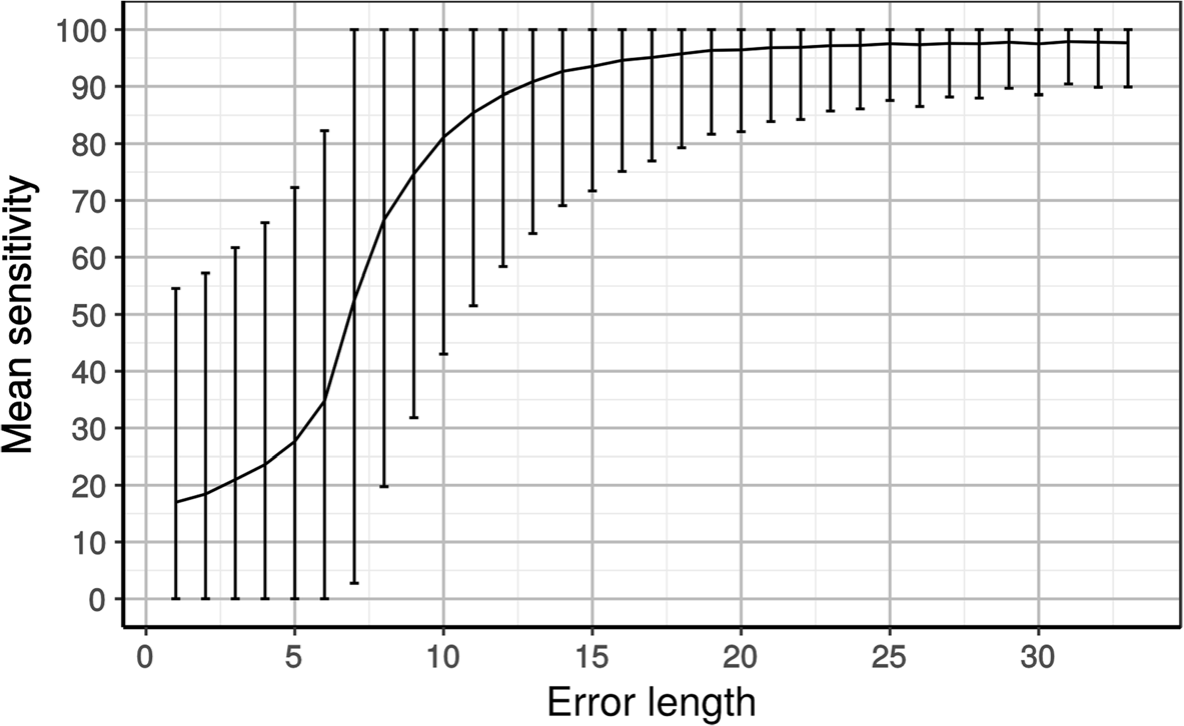
High-resolution analysis of the impact of the length of primary sequence errors on the sensitivity of HmmCleaner used with the default scoring matrix. The plain line represents the improvement in mean sensitivity with increasing error length, while error bars show the variability across 400,000 simulated primary sequence errors in 400 MSAs

To accurately analyze how sensitivity was impacted by error length, more simulations containing a single error 1 to 33 aa in length were carried out with the default scoring matrix of HmmCleaner. As expected, sensitivity increased with error length (Fig. 4), achieving a mean sensitivity < 90% for error lengths < 13 aa. Theoretically, when using the default scoring matrix, an error < 7 aa cannot be detected by HmmCleaner in UARs. This is because this length corresponds to the minimal number of increments needed to decrease the cumulative score from 1 to 0 (− 0.15*7, see Fig. 1). Yet, it is possible to detect shorter errors (e.g., ~ 35% of 6 aa errors) when they are included in divergent regions (AARs) where the score is already < 1. Conversely, the fact that the score is often < 1 increases the probability of reaching 0 by chance, and thus of creating false positives. In other words, HmmCleaner retains some sensitivity for short errors at the expense of its specificity. Importantly, mean sensitivity is > 95% for error lengths > 17 aa, which indicates that only short primary sequence errors will remain in the cleaned MSAs.

HmmCleaner sensitivity proved to be robust to the conservation context of the regions in which primary sequence errors were introduced, except for error lengths < 10 aa (Additional file 1 Figure S6). In particular, short errors were more easily detected in gap-rich regions (Additional file 1 Figure S6A) than in fast-evolving regions (Additional file 1 Figure S6B). In gappy regions, a possible explanation for the good sensitivity could be that there are only few sequences to locally define the pHMM. Consequently, HMMER expects the presence of a highly specific segment of amino acids and is thus more severe when the observed segment does not correspond. Regarding the worse sensitivity in fast-evolving regions, our interpretation is that the pHMM is less specific (flat profile) and can more easily accommodate any divergent segment, including primary sequence errors. In contrast, the level of alignment ambiguity (AAR versus UAR) did not affect the detection of simulated primary sequence errors, whatever the error length (Additional file 1 Figure S6C). HmmCleaner thus accurately detects all simulated errors but shorter ones in all types of regions.

### Overall efficiency of filtering algorithms on primary sequence errors

Having studied the efficiency of segment-filtering methods (HmmCleaner and PREQUAL) only on frameshift primary sequence errors in prokaryotic sequences, it was important to test them on other types of error and other types of sequences. To this end, we considered eukaryotic sequences (112 alignments from mammals and 170 from vertebrates) and two new error types: (i) scrambled amino acid segments generated by shuffling the corresponding underlying individual nucleotides and (ii) arbitrary insertions obtained by inserting shuffled nucleotide segment (see Materials and Methods for details). The sensitivity of HmmCleaner (Table 2) was virtually identical for prokaryotes and eukaryotes, despite very different evolutionary depths (from mammals to euryarchaeotes). Similarly, scrambled segments were detected as efficiently as frameshifts (~ 96%) while arbitrary insertions were more easily recognized (99%). PREQUAL yielded similar results (Table 2), except that its sensitivity was lower for frameshifts in eukaryotes (only 85.64%). Regarding specificity, error type had no effect, except for arbitrary insertions in prokaryotes, which slightly decreased HmmCleaner specificity (85.64% versus ~ 87%), possibly because insertions disturbed the alignment of correct sequences [5]. In contrast, HmmCleaner was more specific for eukaryotic than prokaryotic sequencs (~ 94.5% versus ~ 87%), our eukaryotic genes being less divergent. The same was true for PREQUAL, but to a smaller extent (~ 94.5% versus ~ 92.8%). In summary, sensitivity and specificity of both segment-filtering methods were relatively unaffected by error type and data type. These results were especially welcome for HmmCleaner, which had been trained on prokaryotic frameshifts alone.

**Table 2:** Sensitivity and specificity of filtering software over different error types

sensitivity	prokaryotic MSAs			eukaryotic MSAs		
	frameshifts	scrambled	insertions	frameshifts	scrambled	insertions
HmmCleaner	96.95%	96.24%	99.24%	95.99%	96.26%	99.34%
PREQUAL	93.13%	96.77%	99.83%	85.64%	97.34%	99.95%
BMGE	16.00%	19.23%	97.55%	8.57%	12.57%	96.16%
OD-seq	20.11%	25.09%	59.89%	8.08%	9.34%	59.80%
GUIDANCE2	4.90%	5.37%	0.25%	2.33%	3.25%	1.69%
specificity	prokaryotic MSAs			eukaryotic MSAs		
	frameshifts	scrambled	insertions	frameshifts	scrambled	insertions
HmmCleaner	87.28%	87.24%	85.64%	94.70%	94.70%	94.63%
PREQUAL	92.80%	92.78%	92.76%	94.77%	94.65%	94.59%
BMGE	91.01%	90.88%	91.10%	97.05%	96.93%	96.94%
OD-seq	94.48%	94.46%	92.55%	95.20%	95.25%	94.90%
GUIDANCE2	99.55%	99.48%	99.85%	98.47%	98.35%	98.51%

Segment-filtering methods were developed based on the hypothesis that block-filtering methods are not adapted to detect primary sequence errors. To formally test this assumption, we confronted PREQUAL and HmmCleaner to the block-filtering software BMGE and to OD-seq [24] and GUIDANCE2 [12], two methods designed to filter outlier sequences from MSAs. Our expectations were that block- and outlier-filtering methods would display a limited specificity, even when accurately detecting the simulated errors, due to the former removing the “culprit” segment in all sequences and the latter removing entire sequences. As shown in Table 2, sensitivity of these filtering methods was < 25%, except for arbitrary insertions, for which BMGE was very efficient (~ 97%), and to a lesser extent OD-seq (~ 59%). The performance of BMGE was not surprising, since the insertion of a random segment typically constitutes a divergent block. In contrast, the specificity of block- and outlier-filtering methods (Table 2) was generally higher than the specificity of segment-filtering methods. This was especially true for Guidance (~ 99%), and probably attributable to these methods removing less data than segment-filtering methods. Indeed, as expected from their rationale, methods that filter outlier blocks or outlier sequences appear by design far less sensitive to primary sequence errors than segment filtering methods.

### Sources of HmmCleaner false positives

As shown in Fig. 3, specificity of HmmCleaner is lower than its sensitivity and is also more variable across MSAs. When genuinely homologous segments are highly divergent, i.e., display a weak similarity to other sequences of the MSA, false positives are unavoidable. Accordingly, specificity was higher in UARs (Fig. 2) than in AARs (Additional file 1 Figure S1). A refined analysis shows that HmmCleaner specificity decreases with the gap frequency (Additional file 1 Figure S7A), the evolutionary rate (Additional file 1 Figure S7B) and the fraction of AARs (Additional file 1 Figure S7C). This confirms that its low specificity is due to evolutionary divergence.

Such a negative correlation between sequence divergence and HmmCleaner specificity can be due to: (i) the presence of overlooked primary sequence errors in our datasets, (ii) the presence of alignment errors that would result in detection errors, (iii) the detection of segments corresponding to insertion events, or (iv) the detection of homologous but divergent segments that look like primary sequence errors (see above). For hypothesis one to be true, we should observe approximately the same false positive rate in both UARs and AARs. Yet, this was not the case (Fig. 2 and Additional file 1 Figure S1). Similarly, for hypothesis two to be true, we would expect an important impact of the aligner software on the false positive rate. This was not the case either (Additional file 1 Figure S3). Therefore, the last two hypotheses should explain most of the observed false positives.

**Fig. 5:**
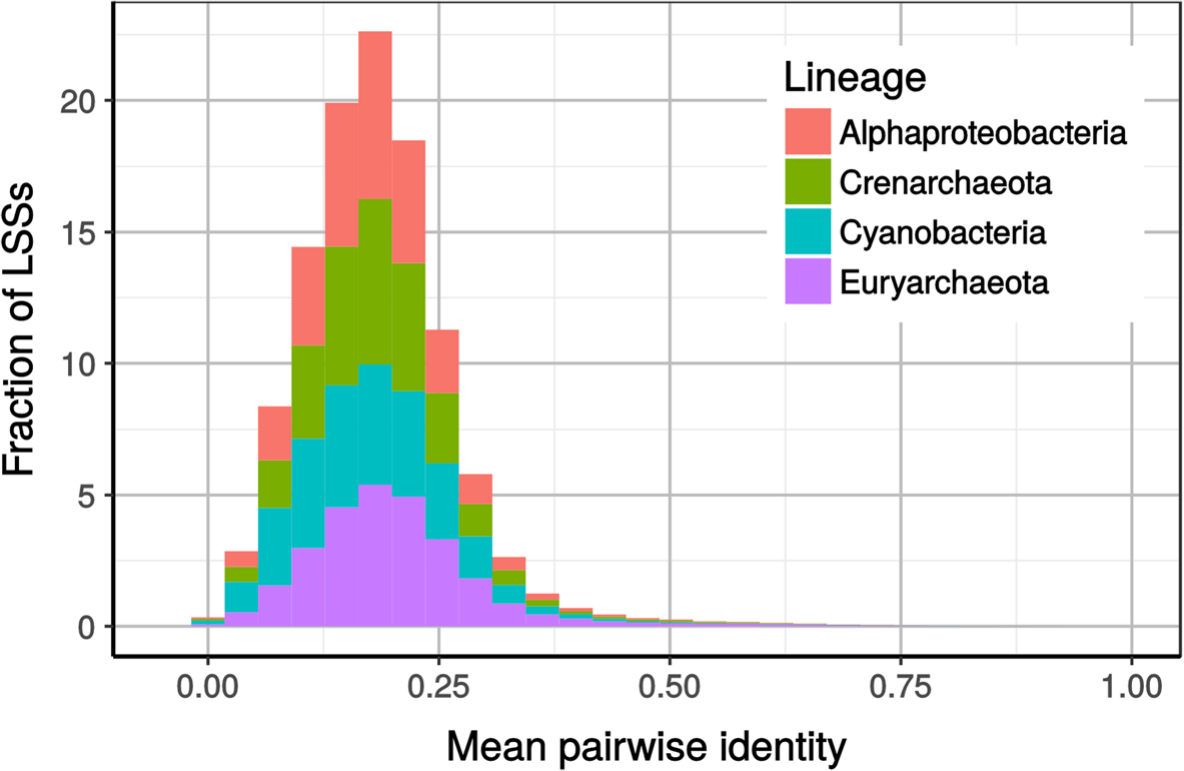
Mean pairwise identity of the low similarity segments (LSSs) detected on 400 raw MSAs (no simulation) by HmmCleaner used with the default scoring matrix

To confirm this interpretation, we ran HmmCleaner on raw MSAs, i.e., without introducing errors, and characterized the segments detected. We considered segments detected in regions with ≥70% of gaps as linked to insertion events. Those segments accounted for 14% of all detected segments. The mean pairwise identity of the remaining segments was mainly distributed between 10 and 30% (Fig. 5), with an average of 19%, indicating HmmCleaner false positives consisted almost exclusively of low similarity segments. Interestingly, the identity window of 10 to 30% is known as the “twilight zone” in structural biology, a zone in which defining homology based on sequence identity alone is hazardous at best. Using known protein structures to define homology, Rost [25] concluded that “above a cut-off roughly corresponding to 30% sequence identity, 90% of the pairs were homologous; below 25% less than 10% were”. Accordingly, the low similarity segments detected by HmmCleaner, even if they do not correspond to true primary sequence errors, are extremely difficult to align and likely contain alignment errors. In contrast, only a small fraction of the segments detected by HmmCleaner (1.8%) had a mean pairwise identity ≥40%. This tiny minority could be considered as the “real” HmmCleaner false positives.

### Detection of positive selection in the presence of primary sequence errors

Having addressed the efficiency of HmmCleaner at dealing with simulated primary sequence errors, we can now assess its usefulness on empirical evolutionary inferences, such as detection of positive selection, topological accuracy and inference of branch lengths. The presence of a primary sequence error in a MSA (i.e., a highly divergent segment) is expected to severely increase the number of non-synonymous substitutions, thereby creating a strong signal for positive selection in the branch leading to the corresponding sequence. To test this idea, we simulated an out-of-frame segment of length 10 to 50 aa into 116 MSAs from the OrthoMAM database, before realigning the MSA (MAMMALIA dataset, see Materials and Methods). Over 10 replicates of the simulation, branch-specific positive selection detected by the standard likelihood ratio test method of Nielsen and Yang [26, 27] increased from 8.28 to 95.69% (Table 3). The few cases with non-significant likelihood ratio test results corresponded to shorter erroneous segments (~ 20 versus ~ 30 aa), which could have been introduced into divergent regions. As expected, a primary sequence error generates a strong erroneous signal of positive selection.

**Table 3:** Detection of positive selection in the presence of primary sequence errors

	Raw	HmmCleaner	PREQUAL	BMGE	TrimAl
MSAs with positive selection in a targeted sequence	8.28%	3.88%	2.67%	7.24%	7.15%
MSAs with positive selection in a targeted sequence with an error	95.69%	7.76%	13.62%	95.00%	94.74%

The use of HmmCleaner on the 116 raw MSAs decreased branch-specific positive selection from 8.28 to 3.88% (Table 3). A detailed manual analysis of the MSAs in which the signal for positive selection had disappeared generally found the presence of structural annotation errors that were correctly detected and removed by HmmCleaner. The remaining 3.88% of significant likelihood ratio test results may correspond to real positive selection or to structural annotation errors not detected by HmmCleaner. Importantly, the use of HmmCleaner on the MSAs in which we had introduced primary sequence errors drastically reduced the detection of positive selection (7.76%). This value was slightly higher than the control (3.88%) and likely due to incomplete removal of the errors by HmmCleaner, in agreement with the sensitivity estimated above (Figs. 3-4). PREQUAL performed similarly to HmmCleaner but was less efficient at reducing the detection of positive selection after error insertion (13.62%), in agreement with its lower sensitivity. In contrast, the use of block-filtering methods (BMGE and TrimAl) had a negligible effect on MSA containing simulated errors (95 and 94.74% respectively, versus 95.69%), which confirms that such methods are not adapted to the removal of primary sequence errors (Table 2).

Nonetheless, our simulations did not allow us to verify that HmmCleaner behaves correctly in real cases of positive selection. To this end, we selected MSAs with well-established presence of sites showing positive selection [27, 28]. Those cases were primate lysozyme c gene, primate cancer gene BRCA1, MHC from human and angiosperm phytochrome genes. Interestingly, neither HmmCleaner nor PREQUAL did remove any residues from primate or human alignments. In contrast, they did on phytochrome genes but subsequent analyses were as significant on the filtered MSAs than on the raw MSAs. In conclusion, both software did not appear to negatively impact detection of true positive selection, at least at a small evolutionary scale.

### Relative effect of primary sequence and alignment errors on phylogenetic inference

To evaluate the relative importance of primary sequence errors and alignment errors on real data, we compared the effect of HmmCleaner, PREQUAL and two block-filtering software (BMGE and TrimAl) on the accuracy of phylogenetic inference. We also examined filtering methods that reduce the stochastic (sampling) error (removal of partial sequences and selection of the longest genes), as this type of error might be critical for single-gene inferences. Two aspects of phylogenetic inference were considered: tree topology and branch lengths. Two datasets of orthologous genes for which the correct species phylogeny is reasonably well established were used: (1) 14,261 genes from mammals, obtained exclusively from genomic data [29], named MAMMALIA, (2) 4593 genes from vertebrates, obtained mainly from transcriptomic data [30], named VERTEBRATA. Our expectation was that primary sequence errors would be more frequent in MAMMALIA than in VERTEBRATA due to incorrect structural annotations of genomic data [31].

Phylogenetic accuracy as measured by the frequency of correctly recovered clades was computed for various conditions (Table 4). Major improvements were observed in three cases: (i) inferences based on nt MSAs over those based on aa MSAs with MAMMALIA (63.19% versus 45.29%), (ii) removal of partial sequences (< 100 aa) in VERTEBRATA (68.70% versus 65.64%) and (iii) use of the longest genes (+ 6.58%, + 8.30% and + 3.21% for nt and aa MAMMALIA and aa VERTEBRATA, respectively). All these cases actually correspond to a reduction of the stochastic error, either due to (i) the increased amount of information present in nt, (ii) the removal of sequences without enough signal to be accurately positioned, or (iii) the larger number of positions, respectively. Stochastic error is therefore the predominant limiting factor for the accuracy of single-gene phylogenies. This is in agreement with Tan et al. [18], who observed that phylogenetic accuracy decreases with the amount of data removed by block-filtering software. This suggests that a filtering method should be highly specific in its removal of erroneous data, otherwise the potential improvement in phylogenetic accuracy could be overthrown by the increase in stochastic error.

**Table 4:** Topological accuracy of single-gene phylogenies

VERTEBRATA		
version	mean	loss (%)
RAW	65.64%	NA
RAW (long)	68.85%	NA
HMM	65.23%	5.61
HMM-L	65.41%	4.22
HMM-LS	65.58%	2.68
PREQUAL	65.73%	3.06
BMGE	64.83%	4.83
TrimAl	65.28%	1.8
HMM Random	64.56%	5.61
HMM + BMGE	63.94%	13.38
HMM + TrimAl	62.90%	13.76
MIN	68.71%	0.71
MIN + HMM	68.67%	6.43
MAMMALIA		
version	mean	loss (%)
RAW (NT)	63.19%	NA
RAW (NT long)	69.77%	NA
HMM (NT)	66.29%	2.92
HMM-L (NT)	66.13%	2.57
HMM-LS (NT)	65.75%	2.12
PREQUAL (NT)	64.77%	2.92
BMGE (NT)	63.59%	3.16
TrimAl (NT)	63.77%	3.76
HMM Random (NT)	62.59%	2.92
HMM + BMGE (NT)	66.56%	4.7
HMM + TrimAl (NT)	66.45%	5.16
RAW (AA)	45.29%	NA
RAW (AA long)	53.59%	NA
HMM (AA)	46.98%	2.92
HMM-L (AA)	46.65%	2.57
HMM-LS (AA)	46.48%	2.12
PREQUAL (AA)	45.63%	2.92
BMGE (AA)	45.45%	3.16
TrimAl (AA)	45.36%	3.76
HMM Random (AA)	44.56%	2.92
HMM + BMGE (AA)	46.52%	4.7
HMM + TrimAl (AA)	46.64%	5.16

^1^mean: average frequency of correctly recovered clades, ^2^loss: fraction of residues removed from the raw MSAs. ^3^long: Values for the half longest MSAs. See the legend of Fig. 6 for the complete set of abbreviations

Generally speaking, the effect of various filtering methods, including HmmCleaner, on phylogenetic accuracy was limited (Table 4). For VERTEBRATA, BMGE, HmmCleaner and TrimAl all slightly decreased accuracy (64.83%, 65.23% and 65.28% versus 65.64%). The performance of HmmCleaner is interesting, because it removes more residues than BMGE and TrimAl (5.6% versus 4.8% and 1.8%, respectively). HmmCleaner thus appears to discard almost exclusively segments that are poorly informative for inferring phylogeny, which is expected because it removes low similarity segments. Accordingly, a random removal of the same amount of data than HmmCleaner decreased accuracy more severely (1.08% versus 0.41%). Moreover, studying the effect of HmmCleaner on each clade of the vertebrate phylogeny reveals that it slightly improved accuracy within clades mainly represented by species for which genomic data had been used (mammals and birds). The use of the large parameter set, which is justified by the presence of > 50 species in VERTEBRATA MSAs, slightly improved accuracy (65.41% versus 65.23% for default parameters), likely because less data were removed (4.22% versus 5.61%). Similarly, the better specificity of PREQUAL (Table 2 and Additional file 1 Table S1) could explain its slightly higher accuracy (65.73%) and the reduced amount of data removal (3.06%). For the VERTEBRATA dataset, which likely contains few primary sequence errors, the removal of data is slightly deleterious (except for PREQUAL, with an improvement of 0.09%), illustrating how precise data filtering should be.

In contrast, the MAMMALIA dataset demonstrated the positive effect of using HmmCleaner on genomic-based datasets, which are more likely to contain annotation errors: accuracy improved from 63.19% to 66.30% for nt MSAs. BMGE and TrimAl also increased accuracy, but less than HmmCleaner (63.59% and 63.77%, respectively), while the random removal of characters expectedly decreased accuracy (62.59%). PREQUAL is in between (64.77%), probably because of its reduced sensitivity (all the more so that structural annotation errors are often shared by unrelated taxa and PREQUAL performed poorly when multiple errors are present in a given alignment (Additional file 1 Table S1). The same pattern was observed for aa sequences (Table 3).

Finally, since segment- and block-filtering methods have different targets (primary sequence and alignment errors, respectively), it could be of interest to combine them, as already done in practice for recent large phylogenomic matrices [30, 32, 33]. To test this, we applied BMGE and TrimAl on the MAMMALIA and VERTEBRATA alignments already cleaned by HmmCleaner. Data loss was important, especially for VERTEBRATA (~ 13.5%), potentially increasing the impact of stochastic error. Accordingly, for VERTEBRATA, the combination of the two types of filters show the lowest accuracy among all our analyses. In contrast, for MAMMALIA, the accuracy increased when both filters were applied versus when a single one was used: from ~ 64.7% to ~ 66.5% (nt) and ~ 45.5% to ~ 46.5% (aa). The comparison of segment+block-filtering with segment-filtering was more ambiguous: the accuracy increased for nt MSAs (from 66.29% for HmmCleaner to 66.56% for HmmCleaner+BMGE, the best accuracy observed for MAMMALIA) but decreased for aa MSAs (from 46.98% for HmmCleaner to 46.52% for HmmCleaner+BMGE). These contrasted results illustrate the difficulty of data filtering, data loss increasing stochastic error while decreasing reconstruction errors.

In conclusion of this section, HmmCleaner is more efficient than BMGE and TrimAl, and to a lesser extent than PREQUAL, at improving topological accuracy for genome-based MSAs, whereas filtering methods slightly decrease accuracy for transcriptome-based MSAs. When primary sequence errors are not negligible, the increase of stochastic error due to data filtering is overcome by the reduction of non-phylogenetic signal. More generally, the better performance of segment-filtering methods (HmmCleaner and PREQUAL) versus block-filtering methods (BMGE and TrimAl) suggests that primary sequence errors (especially annotation errors) are more detrimental to phylogenetic inference than alignment errors.

**Fig. 6:**
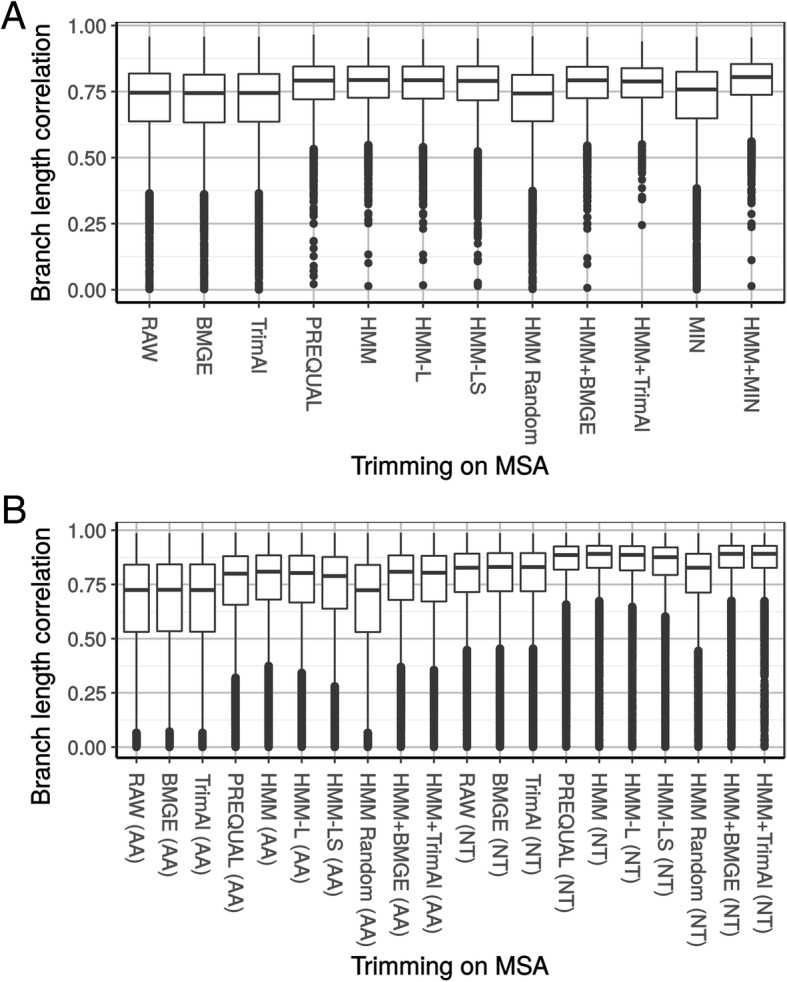
Distribution of correlation coefficients of branch lengths between single gene-tree and the corresponding concatenated tree in different configurations on the VERTEBRATA dataset (**a**) and the MAMMALIA dataset (**b**). RAW: raw MSAs, BMGE: after BMGE with loose settings, TriamAl: after TrimAl in gappy-out configuration, PREQUAL: after PREQUAL, HMM: after HmmCleaner with default preset, HMM-L: after HmmCleaner with large preset, HMM Random: after removing the same number of residues per sequence as HmmCleaner would do but at random, HMM + BMGE: running BMGE after HmmCleaner, HMM + TrimAl: running TrimAl after HmmCleaner, MIN: after removing sequences with < 100 aa, HMM + MIN: combination of HMM then MIN. See Additional file 1: Table S2 for mean values

Since primary sequence errors had only limited (yet detectable) impact on topological accuracy, we wondered if errors could not be “buffered” by the lengthening of the terminal branches leading to the erroneous sequences. To study this possibility, we computed the correlation coefficient between the branch lengths of each single gene and the branch lengths of the concatenated tree, by constraining single-gene trees to the topology of the concatenation. As the genes under study were orthologous, the correlation coefficients were expected to be high [32], fluctuating only because of stochastic sampling noise and heterotachy [34]. For both MAMMALIA and VERTEBRATA aa datasets (Fig. 6), the average correlation coefficients were 0.662 and 0.710, respectively, but 0.778 for MAMMALIA nt MSAs. Again, stochastic error due to the loss of information generated by translation appears as a key factor. In contrast to topological accuracy, the improvement in correlation provided by HmmCleaner was similar for the three datasets (aa VERTEBRATA: 0.066, aa MAMMALIA: 0.089, nt MAMMALIA: 0.079). Interestingly, for MAMMALIA, the average correlation coefficient for cleaned aa MSAs was similar to the one of raw nt MSAs (0.749 and 0.778). PREQUAL performed similarly to HmmCleaner, but was slightly less efficient, even for VERTEBRATA (Fig. 6), likely because of its lowest sensitivity. In sharp contrast, block-filtering methods (BMGE and TrimAl) had virtually no impact, even when applied after HmmCleaner. Segment-filtering methods seem thus to be more efficient than block-filtering methods to remove primary sequence errors affecting branch-length estimates. This is in agreement with our hypothesis that primary sequence errors, which are the target of HmmCleaner and PREQUAL, are more detrimental to evolutionary inferences than alignment errors, which are the target of BMGE and TrimAl.

Finally, we examined the effect of filtering software on the branch lengths of the concatenated trees. All pairwise comparisons (e.g., BMGE versus HmmCleaner) yielded correlation coefficients > 0.98. Interestingly, for VERTEBRATA, a few outliers were identified, corresponding to shortened branch lengths in HmmCleaner-based phylogenies for species from which genomic data had been used (e.g., *Ornithorhynchus*, *Takifugu* or *Latimeria*). We interpret these differences as the result of the removal of structural annotation errors. The negligible impact of filtering software on correlation coefficients in the case of concatenation is likely due to the law of large numbers. In contrast, the tree length (or total branch length) of the concatenated trees was severely modified by all filtering software. For aa supermatrices, the tree length without filtering was 4.46 (14.71) for MAMMALIA (VERTEBRATA). It decreased to 3.85 (11.08) with BGME and to 3.38 (9.07) with HmmCleaner. In agreement with their objectives, this suggests that filtering methods are efficient at removing the more divergent residues that increase tree length. Interestingly, HmmCleaner reduced tree length more than BMGE. As HmmCleaner and BMGE both removed similar numbers of residues (3.1% and 5.6% versus 3.2% and 4.8%, see Table 3), HmmCleaner appears to target divergent residues (due to either primary sequence error or fast evolutionary rate) more efficiently than BMGE.

## Conclusion

In this article, we presented a new version of HmmCleaner, a software package that automatically identifies and removes low similarity segments in MSAs with the purpose of limiting the negative effect of primary sequence errors on evolutionary inferences. The performance of our method was investigated through analyses of both simulated and empirical data. HmmCleaner shows an excellent sensitivity to primary sequence errors ≥12 aa in length in simulations. Its specificity to simulated errors is also high, with its false positives mostly corresponding to insertions or low similarity segments that would be difficult to handle in subsequent steps of analysis.

We showed that segment-filtering software (HmmCleaner and PREQUAL) have more positive effects on evolutionary inferences (detection of branch-specific positive selection, topological accuracy and branch-length estimation) than the commonly used block-filtering software (BMGE and TrimAl). This suggests that primary sequence errors are more detrimental to evolutionary analyses than alignment errors. Therefore, we argue that the efforts of the research community should address both alignment and primary sequence errors, in other words that more energy should be devoted on structural annotations. In this respect, HmmCleaner proved to be efficient at pointing them out, being slightly more sensitive than the recently developed PREQUAL [20].

Given the pervasiveness of primary sequence errors, we recommend the use of segment-filtering methods in high-throughput analyses of eukaryotic genomic data. On the long run, it would be interesting to evaluate whether HmmCleaner (or other equivalent toolssuch as PREQUAL) could replace block-filtering software. For now, HmmCleaner targets low similarity segments that are by essence difficult to align and therefore may decrease the frequency of alignment errors, possibly to the extent of making them negligible. In this respect, the advantage of specifically removing erroneous segments instead of entire blocks is to reduce the amount of data lost for the subsequent analyses, hence limiting the rise in stochastic error, which we have shown to be the major limiting factor for the accuracy of single-gene phylogenies.

## Methods

### HmmCleaner algorithm

HmmCleaner detects low similarity segments in four steps (Fig. 1). First, we create a profile HMM (pHMM) based on the observed data (the MSA) (Fig. 1a). The pHMM is a model of the ancestral sequence that can generate all the observed sequences. It is built with the HMMER function hmmbuild using default options with two exceptions. We change the fragtresh option, giving equal weight to each sequence (−-fragtresh = 0) and we apply a Laplace + 1 prior instead of the default mixture Dirichlet prior (option --plaplace). In our method, the pHMM can either be built upon (i) all sequences of the MSA (complete strategy) or (ii) all sequences except for the one being analyzed (leave-one-out strategy).

Second, we estimate the probability that the pHMM generates each amino acid of a given sequence of the MSA, with the hypothesis that a primary sequence error will have a very low probability. To do so, each sequence of the MSA is evaluated with the pHMM using hmmsearch with default options, which yields profile-sequence alignments (Fig. 1b). HMMER performs this step following a heuristic of homology search at the end of which it defines a set of subsequences (envelopes) estimated to fit a part of the pHMM. Each envelope is then precisely fitted to the profile using the full Forward/Backward algorithm and a maximum expected accuracy alignment is returned [19]. Those alignments allow us to identify which segments of each sequence of the MSA are expected to have been generated by the pHMM, and within each segment, they provide the posterior probability that a specific amino acid has been generated by the pHMM as well as the level of match of each amino acid to the consensus of the pHMM. We used the four discrete categories of match/mismatch defined by HMMER (highlighted in yellow in Fig. 1b), instead of the posterior probability, because preliminary analyses showed that this strategy was more efficient to detect primary sequence errors. This is probably because posterior probability depends both on the quality of the match and on the quality of the alignment around the site while our method focuses solely on the match quality with the assumption that the alignment is correct.

The first two categories defined by HMMER represent residues that do not match the pHMM consensus: blank character (log-odds negative score based on emission probability compared to background frequency) and ‘+’ character (log-odds positive score based on emission probability compared to background frequency, which could be considered a conservative substitution). The last two categories represent residues that match the pHMM consensus: amino acid characters in lowercase (emission probability < 50%) and uppercase (emission probability > 50%). These characters are used to create a string of match/mismatch for HmmCleaner purposes. Segments of this string corresponding to subsequences that do not fit the pHMM (and thus are missing from HMMER output) are filled with blank characters, so as to have a full-length representation of each sequence.

Third, a cumulative similarity score is calculated for each sequence, based on scoring of the four categories of the match/mismatch string. Since we expect that a primary sequence error will mainly consist of mismatches, scoring parameters c1 (blank) and c2 (‘+’) are negative whereas parameters c3 (lower case residue) and c4 (upper case residue) are positive (Fig. 1c). The cumulative similarity score increases when the residue is expected by the pHMM and decreases otherwise (Fig. 1d), representing the evolution of the sequence fit to the pHMM along the sequence. It is computed from left to right, starting at a maximal value of 1, representing a perfect fit to the pHMM, and it is strictly comprised between 0 and 1 included.

Fourth, a low similarity segment is defined wherever the cumulative score reaches zero. Its start is set after the last position where the score was 1, while its end is defined by the last position of the segment where the score was null or by the end of the sequence (Fig. 1d).

### Dataset creation

To optimize the parameters and study the performance of HmmCleaner, we created four datasets by assembling MSAs of protein-coding genes sampled from four different prokaryotic lineages (Alphaproteobacteria, Cyanobacteria, Euryarchaeota, and Crenarchaeota). We chose prokaryotes to minimize the presence of annotation errors, as these lineages are mostly devoid of introns, simplifying the structural annotation of their genes. Yet, a few structural annotation errors will likely subsist, in particular due to sequencing errors, incorrect start codon predictions, programmed ribosomal frameshifts and programmed transcriptional realignments [35]. For each lineage, we retrieved annotated RefSeq genomes from NCBI FTP (61 Alphaproteobacteria, 195 Cyanobacteria, 42 Crenarchaeota and 179 Euryarchaeota) and used the corresponding proteomes to define orthogroups with OrthoFinder (E-value = 10e-5; inflation parameter = 1.5) [36]. In order to maximize the proportion of true orthologous sequences, only orthogroups with at least 75% of our taxon sampling and < 10% of multiple copies were selected. To minimize the sequence length heterogeneity in the orthogroups, we studied the length distribution of the clusters and kept only those having a mean sequence length ≥ 100 aa and with < 5% outliers in the sequence length distribution. Outliers were defined as sequences having a length shorter than the mean length minus 1.96 times the standard deviation. In addition, we removed these outlier sequences from the retained orthogroups. To assemble the four final datasets of 100 MSAs each, we selected at random 100 orthogroups for each of the four lineages, and aligned their sequences with MAFFT 7.309 [21] (L-INS-i algorithm, 5000 iterations). Since our simulations introduce frameshifts in nt sequences (see below), we transferred the alignment gaps from protein sequences to the corresponding nt sequences.

To study the impact of HmmCleaner on evolutionary inferences, we used two additional datasets assembled from animal sequences. The first dataset (MAMMALIA) corresponded to the 14,261 orthologous genes with ≥50% of 43 species present from OrthoMAM v9 [29]. As both nt and amino-acid alignments were available for download, we used both types of sequences. In contrast, only amino-acid sequences were available for the second dataset (VERTEBRATA). The latter corresponded to the 4593 orthologous genes from Irisarri et al. [30]. Because these authors had used filtering softwares during their dataset construction, we had to re-apply their last step (selection of a single sequence per organism and construction of chimeric sequences when necessary) using SCaFoS [37] on a pre-filtering version of the corresponding MSAs.

### Simulator

To study the properties of HmmCleaner, we developed a simulator designed to create primary sequence errors in protein MSAs. In a first step, it takes an existing protein-coding alignment of nt sequences and randomly introduces a primary sequence error in a specified number of sequences. Primary sequence errors can be of three types, (i) a frameshift followed by the opposite (compensatory) mutation after a predefined number of out-of-frame codons, (ii) a scrambled segment resulting from the shuffling of individual nucleotides over a predefined number of codons or (iii) the arbitrary insertion of a segment shuffled as in (ii). Then, it translates all sequences to proteins (ignoring STOP codons, mapped to x characters) and realigns them using MAFFT 7.309 [21] (L-INS-i algorithm, 5000 iterations). In a second step, HmmCleaner is run on the resulting MSA and the detected low similarity segments are compared to the locations of the simulated errors to quantify the number of true positives, false positives, false negatives and true negatives. To allow a fine-grained analysis of the behavior of HmmCleaner, our simulator further characterizes the context of each position of the original MSA by its gap frequency, substitution rate and conservation level, as determined by block-filtering software. More precisely, we used BMGE [9] at three different stringency settings (strict, entropy cutoff of 0.4 and gap cutoff of 0.05; medium, 0.5 and 0.2 corresponding to default parameters; loose, 0.6 and 0.4). As Gblocks [6] yields similar results, only BMGE is considered in this article (data not shown).

### Parameter optimization

To optimize the four parameters of the scoring matrix of HmmCleaner, we simulated frameshift errors on the two large datasets (Cyanobacteria and Euryarchaeota). For each nt MSA, 4 subsets of sequences of different sizes (5, 10, 25 and 50 sequences) were drawn 100 times at random. On each of these samples, 1 to 5 sequences were randomly affected by a primary sequence error of length 10 to 100 aa. HmmCleaner was then run on each resulting amino-acid MSA (complete strategy) under 2835 different combinations of its four parameters. There were 9, 7, 9 and 5 possible values, respectively, for c1 (− 0.05 to − 0.25 by step of 0.025), c2 (− 0.02 to − 0.08 by step of 0.01), c3 (0.05 to 0.25 by step of 0.025) and c4 (0.4 to 0.6 by step of 0.05). These ranges were defined based on preliminary simulations aimed at thoroughly exploring the zone of high specificity and high sensitivity. Moreover, BMGE was run on each simulated MSA to allow a partitioned analysis of the results between ambiguously aligned regions (AARs) and unambiguously aligned regions (UARs).

To ensure that our parameter optimization was robust, we studied the impact of introducing variations in our simulation protocol. First, we focused on a single lineage at a time (either Euryarchaeota or Cyanobacteria) and observed that both sensitivity and specificity computed across the 2835 quartets of parameter values were highly correlated between these two lineages (Pearson correlation coefficient > 0.99, Additional file 1 Figure S2A-B). Second, we compared the results obtained with different operational definitions of UARs. The strictest and the most relaxed configurations of BMGE settings were also highly correlated (> 0.99, Additional file 1 Figure S2C-D). Third, we considered the potential impact of the number of sequences in the MSAs (5, 10, 25 or 50). In this case, we expected a larger effect owing to the dependence of pHMM statistical power on the amount of observations available to build the models. Specificity showed a high correlation (> 0.95) while comparing MSAs with 5 sequences to those with 50 sequences (Additional file 1 Figure S2F). In contrast, sensitivity was more affected, and the correlation coefficient dropped to 0.78 (Additional file 1 Figure S2E). In agreement with our intuition, sensitivity was always better with 50 than with 5 sequences. However, the parameter quartets leading to high sensitivity for 5 sequences were the same to yield high sensitivity for 50 sequences, indicating that the number of species does not much impact the optimization of HmmCleaner parameters.

### Characterization of HmmCleaner performance

To characterize the impact of the length and number of frameshift errors on HmmCleaner sensitivity and specificity, we ran simulations at 4 predetermined error lengths (10, 33, 66 and 100 aa) and with 4 different numbers of sequences affected (1, 5, 10 or 15). For each of these 16 combinations, 100 simulations were performed on each of the 100 MSAs of the 4 lineages using subsets of 25 randomly drawn sequences. HmmCleaner was run on these 160,000 MSAs per prokaryotic lineage (640,00 MSAs in total) using the default scoring matrix and the complete strategy. Ten additional simulations were carried out in the same conditions (16 combinations of error characteristics on subsets of 25 sequences) to compare HmmCleaner with the four scoring matrix and PREQUAL. PREQUAL was run with default parameters and without the removal of repeated regions. For the high-resolution analysis of the impact of the error length on sensitivity, the same type of simulation was run with only one sequence affected by a primary sequence error of length ranging from 1 to 33 aa.

Additional simulations were carried out to expand the observations obtained on frameshift errors to the other types of primary sequence errors that our simulator can generate. At the same time, we extended our dataset to MSAs of eukaryotic species by selecting 112 alignments from the MAMMALIA dataset (MSA with > 25 sequences out of the 116 genes used for positive selection, see below) and 170 alignments from VERTEBRATA for which we retrieved nucleotide sequences (MSAs with less than 3 missing species). For each type of errors (frameshifts, scrambled segments and arbitrary insertions), subsets of 25 sequences were drawn out of the complete MSA and 1 to 5 sequences were affected by an error of length between 10 to 100 aa. This simulation was run 100 times per MSA for HmmCleaner (default), BMGE (loose) and PREQUAL, and 10 times for OD-SEQ (default parameters) and GUIDANCE2 (default threshold to determine outlier sequences).

To study the characteristics of the low similarity segments detected by HmmCleaner, so as to characterize the sources of its false positives, it was run with the default scoring matrix and the complete strategy on the raw MSAs from the four prokaryotic lineages, as MSAs of eukaryotic lineages are more likely to contain real primary sequence errors (mainly incorrect structural annotation). For each detected segment, we computed the gap frequency in the corresponding region of the MSA and its mean pairwise identity. Pairwise identity itself was considered as computable when ≥10% of the low similarity segment residues were facing a residue (and not a gap) in the opposite sequence. Likewise, mean pairwise identity was computed only when ≥10% of the pairs were computable.

### Effect of HmmCleaner on evolutionary inferences

Analyses of positive selection were performed on a subset of the MAMMALIA dataset. To reduce structural annotation errors, we selected the 446 nt MSAs with branch length R^2^ above 0.95 (computed as in Simion et al. 2017 [32], see below). To limit the computational burden and to introduce errors of a sizable length representing only a few percent of the sequences, we selected the 116 MSAs between the first quartile and the median on the MSA width distribution. For each MSA, we simulated one primary sequence error of random length (10 to 50 aa) at a randomly chosen position of a randomly chosen sequence 10 times and aligned with MAFFT. For each simulation, we tested for positive selection in the affected branch for 10 versions of the corresponding MSA: (1) the original MSA, (2) the original MSA cleaned by 5 filtering software configurations (HmmCleaner, PREQUAL, BMGE and TrimAl), (3) the erroneous MSA, and (4) the erroneous MSA cleaned by 5 filtering software configurations (HmmCleaner, PREQUAL, BMGE and TrimAl). Detection of positive selection was performed using a likelihood ratio test between two models [26, 27]: model A, in which ω estimation is free (i.e., allowing positive selection), and model B, in which ω is fixed to 1 (i.e., no selection). Likelihood values for both models were obtained using codeml (both models: runmode = 0, method = 0, clock = 0, model = 2, CodonFreq = 2, NSsites = 2, fix_kappa = 0, kappa = 2; model A: fix_omega = 0, omega = 0.2; model B: fix_omega = 1, omega = 1). Positive selection was considered present when the likelihood ratio test between models A and B returned a value > 13.82 (Chi-square critical value for alpha = 0.001 and 2 degrees of freedom).

To test the effect of filtering methods on the accuracy of single-gene phylogenies, we used the orthologous genes of the datasets MAMMALIA and VERTEBRATA. Eleven different filtering setups were considered: (1) RAW: without any alteration the MSA, (2) HMM: HmmCleaner with the default scoring matrix (for nt MSA, low similarity segments were detected on the corresponding protein MSAs and then reported), (3) HMM-L: HmmCleaner with the “large” scoring matrix, (4) PREQUAL: PREQUAL with default parameters, (5) BMGE: BMGE in loose settings, (6) TrimAl: TrimAl with gappy-out option, (7) HMM Random: removal of the same number of residues per sequence as HmmCleaner would have done but at random, (8) HMM + BMGE: running BMGE as in 5 after HmmCleaner, (9) HMM + TrimAl: running TrimAl as in 6 after HmmCleaner, (10) MIN: removal of the sequences with < 100 residues and (11) HMM + MIN: a combination of running HmmCleaner then removing sequences as in MIN.

Single-gene trees were inferred with RAxML v8 [38] with the PROTGAMMALGF model for protein MSAs and the GTRGAMMA model for nt MSAs. Frequencies of correctly recovered clades were computed with a custom script comparing the single-gene trees to the topology of Irisarri et al. [30] for the VERTEBRATA dataset and to a concatenated tree of the 137 most complete aa MSAs inferred with PhyloBayes-MPI [39] using the CAT+G model [40] for the MAMMALIA dataset. These two topologies are in agreement with existing knowledge of vertebrate relationships, even if ambiguities persist for a few nodes (e.g., the relative position of Xenarthra and Afrotheria).

For each MSA, branch lengths were computed with RAxML using the same model as previously while constraining the topology to the respective reference species tree. Single-gene branch lengths were then compared to the branch lengths of the reference tree, after pruning the species missing in the MSA under study. Finally, the correlation coefficient of the two sets of branch lengths was computed with a custom script.

## Additional file

Additional file 1: **Table S1.** Comparison of mean sensitivity and mean specificity between HmmCleaner presets and PREQUAL. **Table S2. Mean R2 value for branch length**. **Figure S1.** Mean sensitivity and specificity of HmmCleaner towards detection of primary sequence errors introduced in ambiguously aligned regions (AARs). **Figure S2.** Impact of the conditions of simulation on sensitivity (A,C,E) and specificity (B,D,F) of HmmCleaner. **Figure S3.** Impact of the multiple alignment software on sensitivity (A) and specificity (B) of HmmCleaner used with the default scoring matrix. **Figure S4.** Impact of the HmmCleaner algorithm on its sensitivity (A) and specificity (B) when used with the default scoring matrix. **Figure S5.** Impact of the length and number of primary sequence errors, and of the prokaryotic lineage, on sensitivity (A,C,E) and specificity (B,D,F) of PREQUAL. A,B. **Figure S6.** Impact of the conservation context of introduced primary sequence errors on sensitivity of HmmCleaner used with the default scoring matrix for different error lengths. **Figure S7.** Impact of the conservation context of introduced primary sequence errors on specificity of HmmCleaner used with the default scoring matrix for different numbers of errors. (PDF 1324 kb)

## Abbreviations

aa: Amino acids
AAR: Ambiguously aligned region
LRT: Likelihood ratio test
MSA: Multiple sequences alignment
nt: Nucleotides
pHMM: Profile hidden Markov model
UAR: Unambiguously aligned region

## References

[1] Chatzou M, Magis C, Chang J-M, Kemena C, Bussotti G, Erb I (2016). Multiple sequence alignment modeling: methods and applications. Brief Bioinform.

[2] Wong KM, Suchard MA, Huelsenbeck JP. Alignment uncertainty and genomic analysis. Science (80-. ). 2008;319:473–476. 10.1126/science.1151532 18218900

[3] Philippe H, Brinkmann H, Lavrov DV, Littlewood DTJ, Manuel M, Wörheide G, et al. Resolving difficult phylogenetic questions: why more sequences are not enough. PLoS Biol. 2011;9. 10.1371/journal.pbio.1000602 PMC3057953 21423652

[4] Schneider A, Souvorov A, Sabath N, Landan G, Gonnet GH, Graur D (2009). Estimates of positive Darwinian selection are inflated by errors in sequencing, annotation, and alignment. Genome Biol Evol.

[5] Markova-Raina P, Petrov D (2011). High sensitivity to aligner and high rate of false positives in the estimates of positive selection in the 12 Drosophila genomes. Genome Res.

[6] Castresana J (2000). Selection of conserved blocks from multiple alignments for their use in phylogenetic analysis. Mol Biol Evol.

[7] Dress AW, Flamm C, Fritzsch G, Grünewald S, Kruspe M, Prohaska SJ (2008). Noisy: identification of problematic columns in multiple sequence alignments. Algorithms Mol Biol.

[8] Capella-Gutiérrez S, Silla-Martínez JM, Gabaldón T (2009). trimAl: a tool for automated alignment trimming in large-scale phylogenetic analyses. Bioinformatics.

[9] Criscuolo A, Gribaldo S (2010). BMGE (block mapping and gathering with entropy): a new software for selection of phylogenetic informative regions from multiple sequence alignments. BMC Evol Biol.

[10] Kück P, Meusemann K, Dambach J, Thormann B, von Reumont BM, Wägele JW (2010). Parametric and non-parametric masking of randomness in sequence alignments can be improved and leads to better resolved trees. Front Zool.

[11] Wu M, Chatterji S, Eisen JA (2012). Accounting for alignment uncertainty in phylogenomics. PLoS One.

[12] Sela I, Ashkenazy H, Katoh K, Pupko T (2015). GUIDANCE2: accurate detection of unreliable alignment regions accounting for the uncertainty of multiple parameters. Nucleic Acids Res. Oxford University Press.

[13] Talavera G, Castresana J (2007). Improvement of phylogenies after removing divergent and ambiguously aligned blocks from protein sequence alignments. Syst Biol.

[14] Jordan G, Goldman N (2012). The effects of alignment error and alignment filtering on the sitewise detection of positive selection. Mol Biol Evol.

[15] Privman E, Penn O, Pupko T (2012). Improving the performance of positive selection inference by filtering unreliable alignment regions. Mol. Biol. Evol. Oxford University Press.

[16] Karin EL, Susko E, Pupko T (2014). Alignment errors strongly impact likelihood-based tests for comparing topologies. Mol Biol Evol.

[17] Spielman SJ, Dawson ET, Wilke CO (2014). Limited utility of residue masking for positive-selection inference. Mol Biol Evol.

[18] Tan G, Muffato M, Ledergerber C, Herrero J, Goldman N, Gil M (2015). Current methods for automated filtering of multiple sequence alignments frequently worsen single-gene phylogenetic inference. Syst Biol.

[19] Eddy SR. Accelerated profile HMM searches. PLoS Comput Biol. 2011;7. 10.1371/journal.pcbi.1002195 PMC3197634 22039361

[20] Whelan S, Irisarri I, Burki FPREQUAL. Detecting non-homologous characters in sets of unaligned homologous sequences. Bioinformatics. 2018:1–2. 10.1093/bioinformatics/bty448 29868763

[21] Katoh K, Standley DM (2013). MAFFT multiple sequence alignment software version 7: improvements in performance and usability. Mol Biol Evol.

[22] Edgar RC (2004). MUSCLE: multiple sequence alignment with high accuracy and high throughput. Nucleic Acids Res.

[23] Sievers F, Wilm A, Dineen D, Gibson TJ, Karplus K, Li W (2011). Fast, scalable generation of high-quality protein multiple sequence alignments using Clustal omega. Mol Syst Biol.

[24] Jehl P, Sievers F, Higgins DG (2015). OD-seq: outlier detection in multiple sequence alignments. BMC bioinformatics. BioMed Central.

[25] Rost B (1999). Twilight zone of protein sequence alignments. Protein Eng Des Sel.

[26] Nielsen R, Yang Z (1998). Likelihood models for detecting positively selected amino acid sites and applications to the HIV-1 envelope gene. Genetics.

[27] Zhang J, Nielsen R, Yang Z (2005). Evaluation of an improved branch-site likelihood method for detecting positive selection at the molecular level. Mol Biol Evol.

[28] Yang Z, Swanson WJ (2002). Codon-substitution models to detect adaptive evolution that account for heterogeneous selective pressures among site classes. Mol Biol Evol.

[29] Ranwez V, Delsuc F, Ranwez S, Belkhir K, Tilak MK, Douzery EJP (2007). OrthoMaM: a database of orthologous genomic markers for placental mammal phylogenetics. BMC Evol Biol.

[30] Irisarri I, Baurain D, Brinkmann H, Delsuc F, Sire JY, Kupfer A (2017). Phylotranscriptomic consolidation of the jawed vertebrate timetree. Nat Ecol Evol.

[31] Sharma V, Hiller M (2017). Increased alignment sensitivity improves the usage of genome alignments for comparative gene annotation. Nucleic Acids Res Oxford University Press.

[32] Simion P, Philippe H, Baurain D, Jager M, Richter DJDJ, Di Franco A (2017). A large and consistent Phylogenomic dataset supports sponges as the sister group to all other animals. Curr Biol.

[33] Amemiya CT, Alföldi J, Lee AP, Fan S, Philippe H, MacCallum I (2013). The African coelacanth genome provides insights into tetrapod evolution. Nature Nature Publishing Group.

[34] Lopez P, Casane D, Philippe H (2002). Heterotachy, an important process of protein evolution. Mol Biol Evol Oxford University Press.

[35] Sharma V, Firth AE, Antonov I, Fayet O, Atkins JF, Borodovsky M (2011). A pilot study of bacterial genes with disrupted ORFs reveals a surprising profusion of protein sequence recoding mediated by ribosomal frameshifting and transcriptional realignment. Mol Biol Evol.

[36] Emms DM, Kelly S (2015). OrthoFinder: solving fundamental biases in whole genome comparisons dramatically improves orthogroup inference accuracy. Genome Biol.

[37] Roure B, Rodriguez-Ezpeleta N, Philippe H (2007). SCaFoS: a tool for selection, concatenation and fusion of sequences for phylogenomics. BMC Evol Biol.

[38] Stamatakis A (2014). RAxML version 8: a tool for phylogenetic analysis and post-analysis of large phylogenies. Bioinformatics.

[39] Lartillot N, Rodrigue N, Stubbs D, Richer J (2013). PhyloBayes MPI : phylogenetic reconstruction with infinite mixtures of profiles in a parallel environment. Syst Biol.

[40] Lartillot N, Philippe HA (2004). Bayesian mixture model for across-site heterogeneities in the amino-acid replacement process. Mol Biol Evol.

[41] Philippe H (1993). MUST, a computer package of management utilities for sequences and trees. Nucleic Acids Res.

