# Supplementary material for: Evaluating the usefulness of alignment filtering methods to reduce the impact of errors on evolutionary inferences
Source: BMC Evol Biol. 2019 Jan 11;19:21. doi: 10.1186/s12862-019-1350-2 (PMC6330419; doi:10.1186/s12862-019-1350-2)
Supplement: Supplementary file 1 — Table S1. Comparison of mean sensitivity and mean specificity between HmmCleaner presets and PREQUAL. Table S2. Mean R2 value for branch length. Figure S1. Mean sensitivity and specificity of HmmCleaner towards detection of primary sequence errors introduced in ambiguously aligned regions (AARs). Figure S2. Impact of the conditions of simulation on sensitivity (A,C,E) and specificity (B,D,F) of HmmCleaner. Figure S3. Impact of the multiple alignment software on sensitivity (A) and specificity (B) of HmmCleaner used with the default scoring matrix. Figure S4. Impact of the HmmCleaner algorithm on its sensitivity (A) and specificity (B) when used with the default scoring matrix. Figure S5. Impact of the length and number of primary sequence errors, and of the prokaryotic lineage, on sensitivity (A,C,E) and specificity (B,D,F) of PREQUAL. A,B. Figure S6. Impact of the conservation context of introduced primary sequence errors on sensitivity of HmmCleaner used with the default scoring matrix for different error lengths. Figure S7. Impact of the conservation context of introduced primary sequence errors on specificity of HmmCleaner used with the default scoring matrix for different numbers of errors. (PDF 1324 kb) [file 12862_2019_1350_MOESM1_ESM.pdf]

Supplementary Table 1. Comparison of mean sensitivity and mean specificity between HmmCleaner presets and PREQUAL

**Sensibility**	Overall	Error length				Error number				Lineage			
		10	33	66	100	1	5	10	15	Alpha-proteobacteria	Crenarcheota	Cyanobacteria	Euryarcheota
PREQUAL	83.33% (15.92)	60.72% (10.36)	91.34% (6.23)	91.70% (8.26)	89.56% (10.73)	87.68% (17.44)	85.63% (15.05)	81.90% (14.50)	78.11% (14.84)	84.76% (14.77)	83.82% (16.69)	79.72% (16.96)	85.02% (14.56)
Default	93.30% (9.11)	80.35% (8.86)	97.36% (3.04)	97.80% (3.06)	97.67% (3.36)	91.7% (11.04)	93.75% (8.38)	93.89% (8.23)	93.84% (8.30)	92.90% (8.78)	93.52% (8.84)	92.69% (10.64)	94.07% (7.91)
Large	90.16% (11.07)	74.16% (9.28)	95.81% (3.82)	95.85% (4.34)	94.80% (5.36)	88.32% (12.58)	90.28% (10.59)	90.96% (10.32)	91.06% (10.42)	89.05% (10.62)	90.66% (10.93)	90.24% (12.36)	90.67% (10.16)
Specificity	86.10% (17.41)	58.49% (11.76)	94.93% (3.99)	95.69% (4.27)	95.27% (4.93)	84.65% (17.87)	86.36% (17.26)	86.68% (17.20)	86.69% (17.25)	84.89% (16.51)	86.52% (17.55)	85.59% (19.55)	87.38% (15.71)
Large_ Specificity	76.20% (26.59)	32.96% (12.47)	90.65% (6.76)	91.64% (7.22)	89.55% (9.00)	73.77% (26.37)	76.23% (26.08)	77.19% (26.80)	77.63% (26.98)	73.94% (24.39)	77.15% (27.31)	76.61% (29.31)	77.12% (24.97)
**Specificity**	Overall	Error length				Error number				Lineage			
		10	33	66	100	1	5	10	15	Alpha-proteobacteria	Crenarcheota	Cyanobacteria	Euryarcheota
PREQUAL	92.42% (6.34)	93.83% (6.09)	92.44% (6.31)	92.26% (6.44)	92.16% (6.51)	92.99% (6.00)	92.67% (6.18)	92.24% (6.41)	91.80% (6.71)	90.28% (8.25)	92.61% (6.04)	94.99% (4.15)	91.82% (5.26)
Default	86.70% (10.28)	87.13% (9.93)	86.97% (10.04)	86.62% (10.33)	86.07% (10.80)	87.64% (9.81)	87.01% (10.10)	86.36% (10.41)	85.79% (10.70)	86.23% (11.08)	85.39% (11.41)	90.88% (7.46)	84.29% (9.46)
Large	90.98% (7.37)	91.40% (7.02)	91.23% (7.12)	90.87% (7.43)	90.41% (7.84)	91.78% (6.92)	91.23% (7.22)	90.69% (7.45)	90.21% (7.77)	90.20% (7.95)	90.46% (8.32)	93.72% (5.29)	89.53% (6.79)
Specificity	91.80% (6.99)	92.13% (6.73)	91.96% (6.82)	91.71% (7.02)	91.38% (7.34)	92.48% (6.63)	92.03% (6.85)	91.56% (7.05)	91.12% (7.33)	91.39% (7.29)	91.23% (8.07)	94.14% (5.14)	90.41% (6.53)
Large_ Specificity	94.65% (4.88)	95.00% (4.61)	94.72% (4.77)	94.55% (4.93)	94.33% (5.19)	95.18% (4.57)	94.85% (4.75)	94.47% (4.94)	94.09% (4.75)	94.11% (5.01)	94.51% (5.78)	96.13% (3.61)	93.86% (4.56)

Supplementary Table 2. Mean R2 value for branch length

VERTEBRATA	
version	mean R2 BL
RAW	0.709
BMGE	0.705
TrimAl	0.707
PREQUAL	0.770
HMM	0.775
HMM-L	0.773
RANDOM	0.705
HMM+BMGE	0.773
HMM+TrimAl	0.771
MIN	0.716
MIN+HMM	0.787

MAMMALIA	
version	mean R2 BL
RAW (AA)	0.660
BMGE (AA)	0.662
TrimAl (AA)	0.660
PREQUAL (AA)	0.736
HMM (AA)	0.749
HMM-L (AA)	0.740
HMM Random (AA)	0.659
HMM+BMGE (AA)	0.748
HMM+TrimAl (AA)	0.743
RAW (NT)	0.776
BMGE (NT)	0.778
TrimAl (NT)	0.777
PREQUAL (NT)	0.849
HMM (NT)	0.855
HMM-L (NT)	0.844
HMM Random (NT)	0.775
HMM+BMGE (NT)	0.855
HMM+TrimAl (NT)	0.856

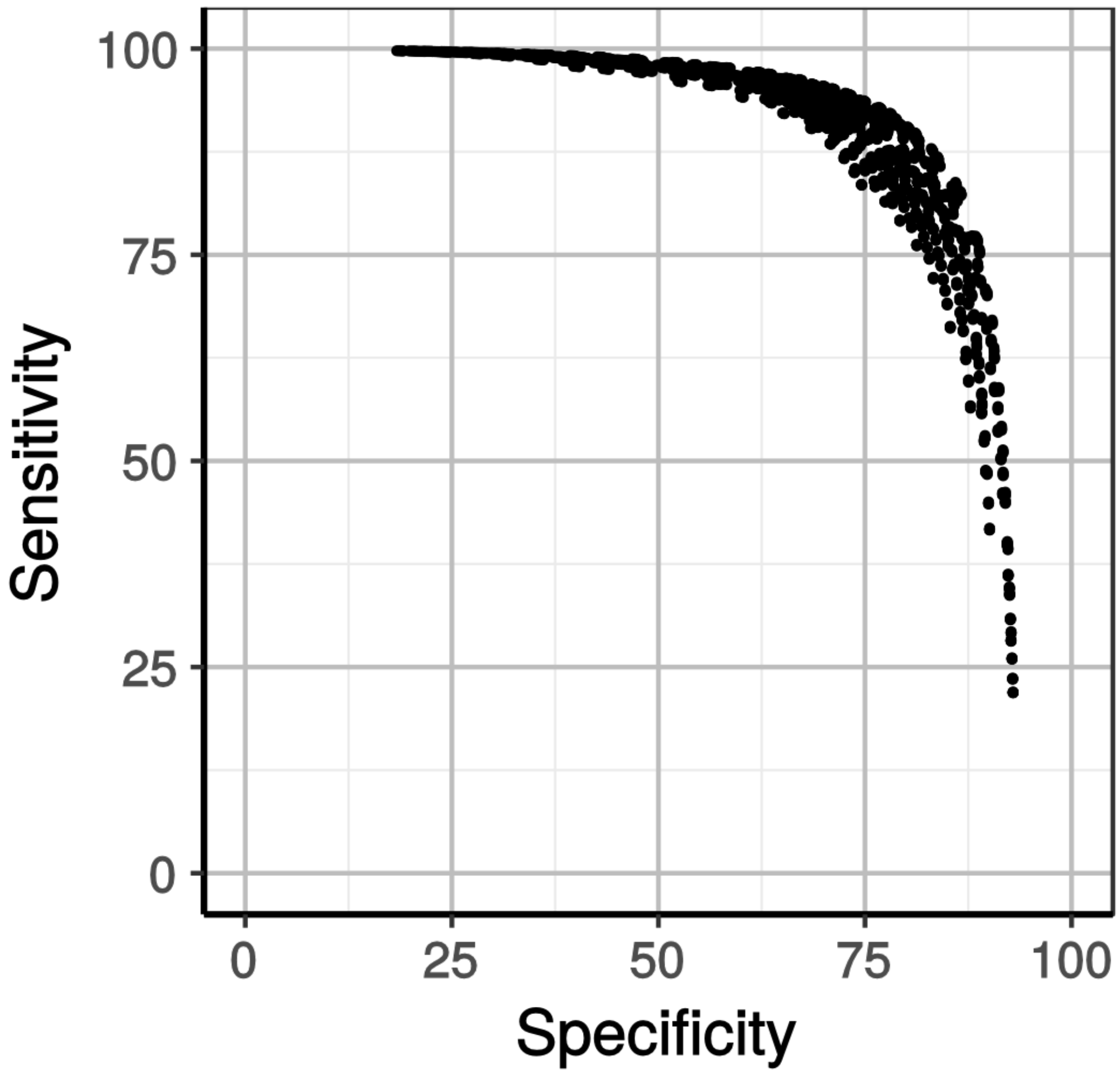

Suppl. Figure 1

Mean sensitivity and specificity of HmmCleaner towards detection of primary sequence errors introduced in ambiguously aligned regions (AARs). Each dot corresponds to the two means of the values obtained across 80,000 simulations and 3 operational definitions of AARs for one of the 2835 combinations of the 4 parameters of the scoring matrix.

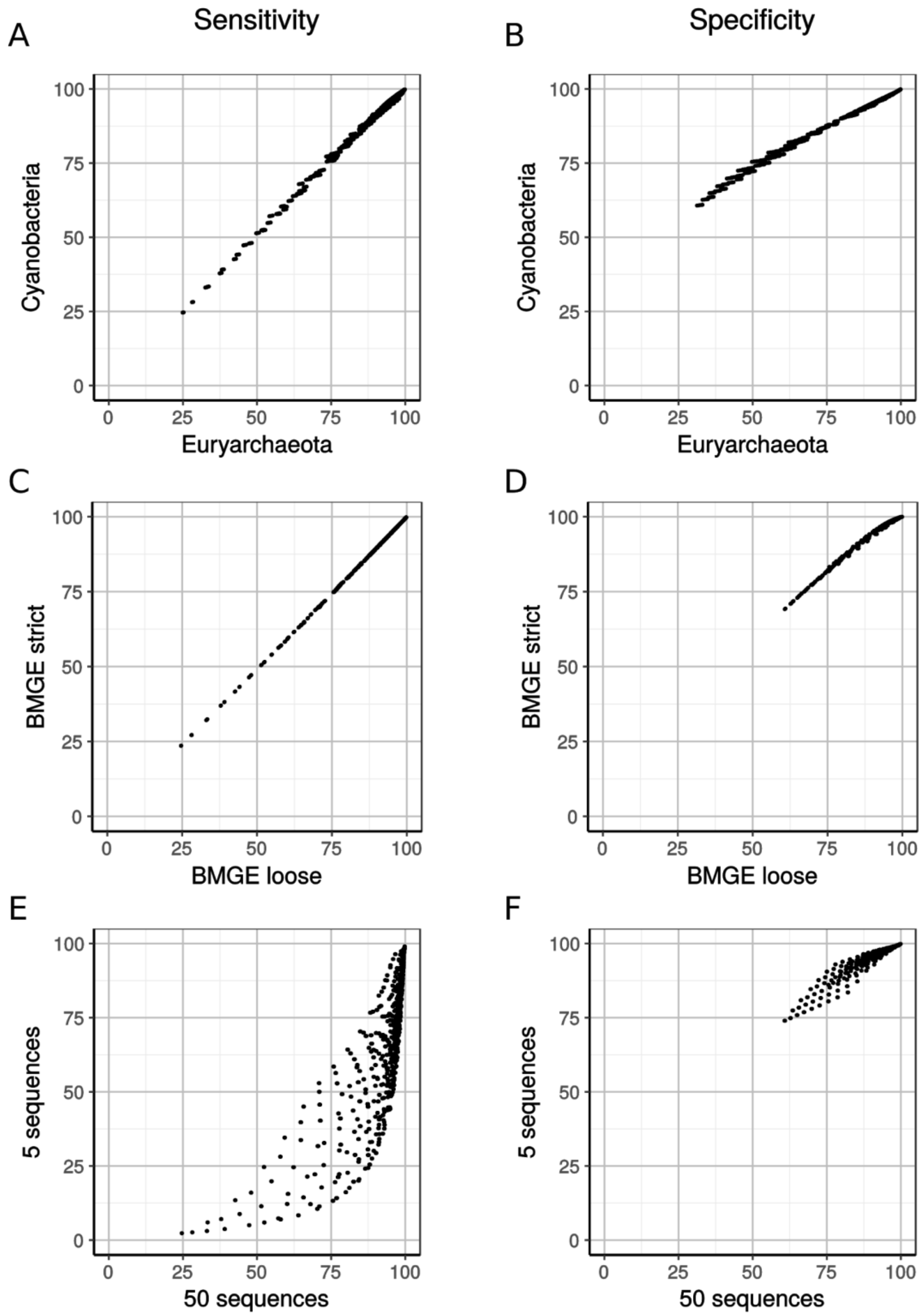

Suppl. Figure 2

Impact of the conditions of simulation on sensitivity (A,C,E) and specificity (B,D,F) of HmmCleaner. A,B. Comparison between simulations using MSAs from Euryarchaeota and Cyanobacteria. C,D. Comparison between determination of UARs with BMGE using loose and strict settings. E,F. Comparison between simulations using MSAs of 5 and 50 sequences.

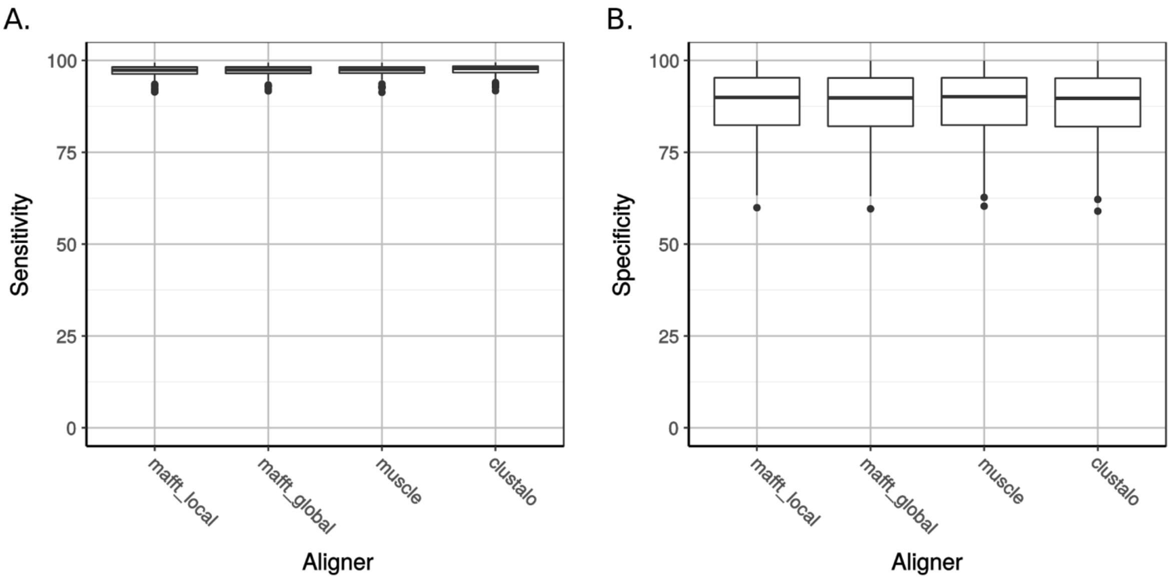

Suppl. Figure 3

Impact of the multiple alignment software on sensitivity (A) and specificity (B) of HmmCleaner used with the default scoring matrix. The compared aligners were MAFFT with L-INS-i algorithm (mafft_local, default aligner), MAFFT with G-INS-i algorithm (mafft_global), MUSCLE and Clustal Omega. Simulations were run on MSAs with 25 species (either Alpha-proteobacteria or Crenarchaeota), by introducing 1 to 5 primary sequence errors of length 10 to 100 aa, and both AARs and UARs were considered when computing the statistics. Box-plots were computed across all considered MSAs.

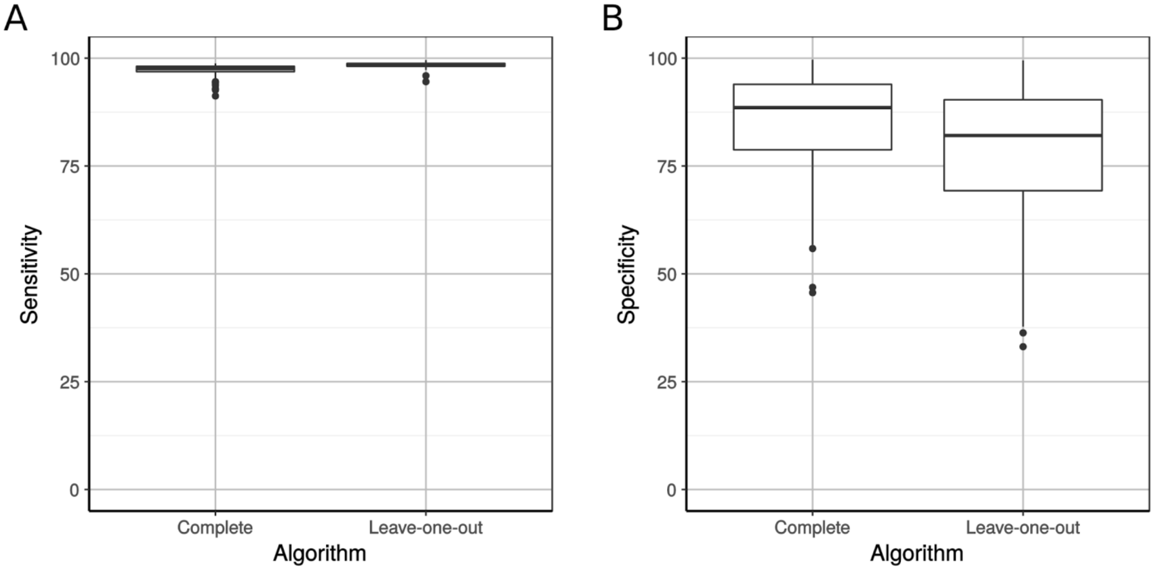

Suppl. Figure 4

Impact of the HmmCleaner algorithm on its sensitivity (A) and specificity (B) when used with the default scoring matrix. The compared algorithms were the "complete strategy" (using all sequences to build the pHMM) and the "leave-one-out strategy" (using all sequences but the analyzed one). Simulations were run on MSAs with 25 species (either Alpha-proteobacteria or Crenarchaeota), by introducing 4 predetermined numbers of primary sequence errors (1, 5, 10 and 15) of 4 specific lengths (10, 33, 66 and 100 aa), and both AARs and UARs were considered when computing the statistics. Box-plots were computed across all considered MSAs.

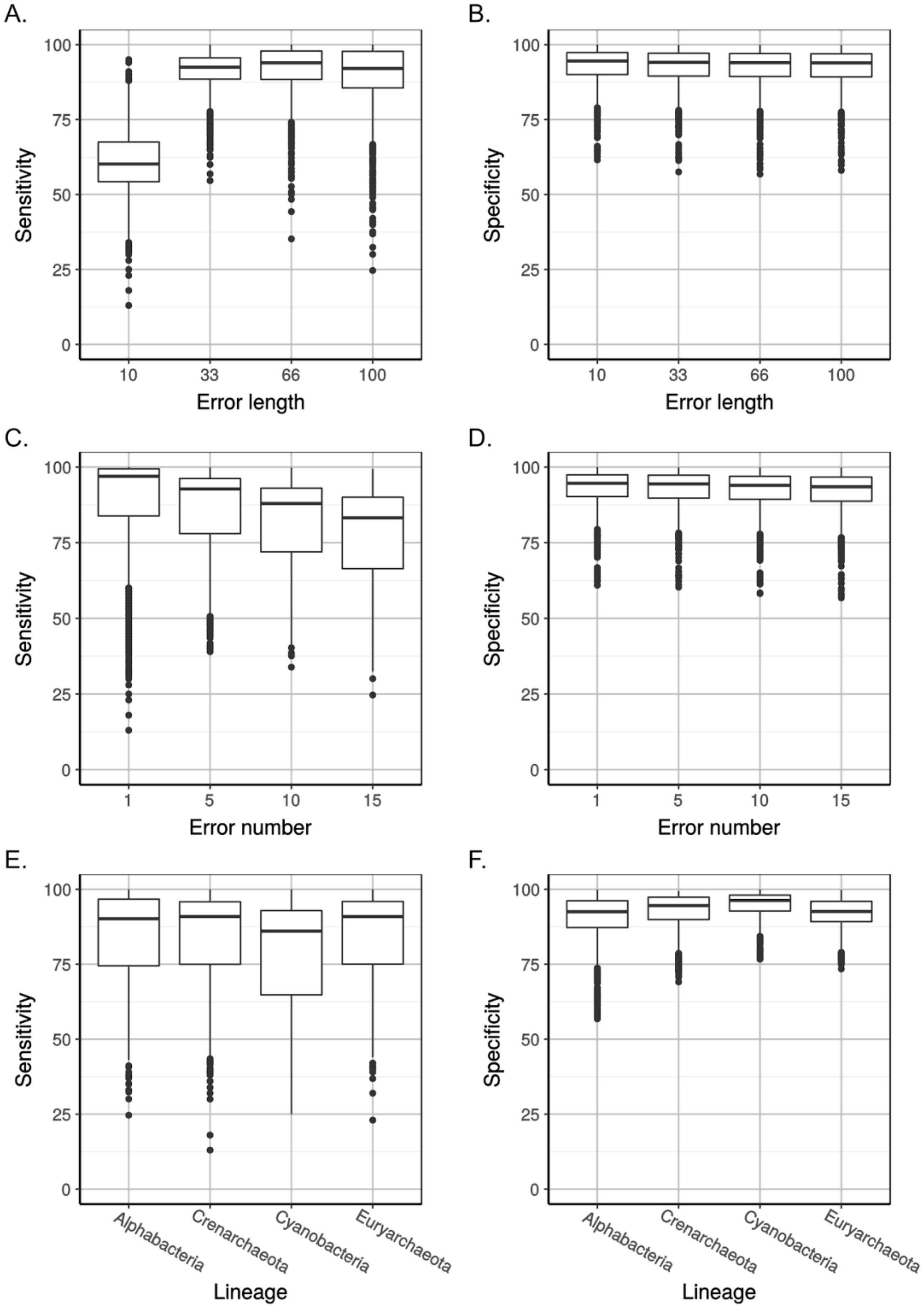

Suppl. Figure 5

Impact of the length and number of primary sequence errors, and of the prokaryotic lineage, on sensitivity (A,C,E) and specificity (B,D,F) of PREQUAL. A,B. Effect of primary sequence error length. C,D. Effect of the number of primary sequence errors. E,F. Effect of the prokaryotic lineage. Box-plots were computed across all considered MSAs and values are means averaged over the different conditions of simulation.

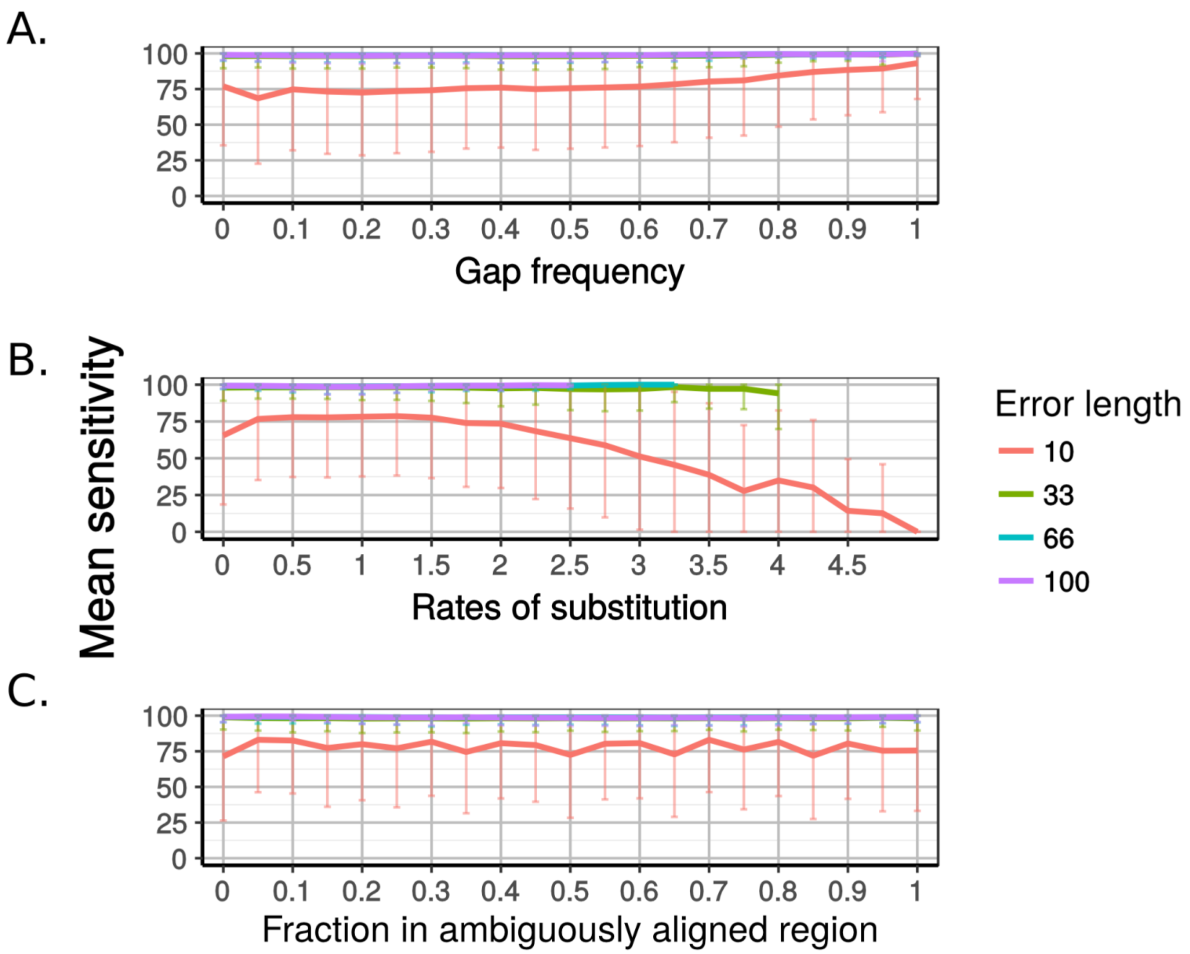

Suppl. Figure 6

Impact of the conservation context of introduced primary sequence errors on sensitivity of HmmCleaner used with the default scoring matrix for different error lengths. A. Sensitivity relative to the mean gap frequency in the region of insertion. B. Sensitivity relative to the mean rate of substitution in the region of insertion. C. Sensitivity relative to the fraction of the region of insertion defined as AAR by BMGE (loose settings).

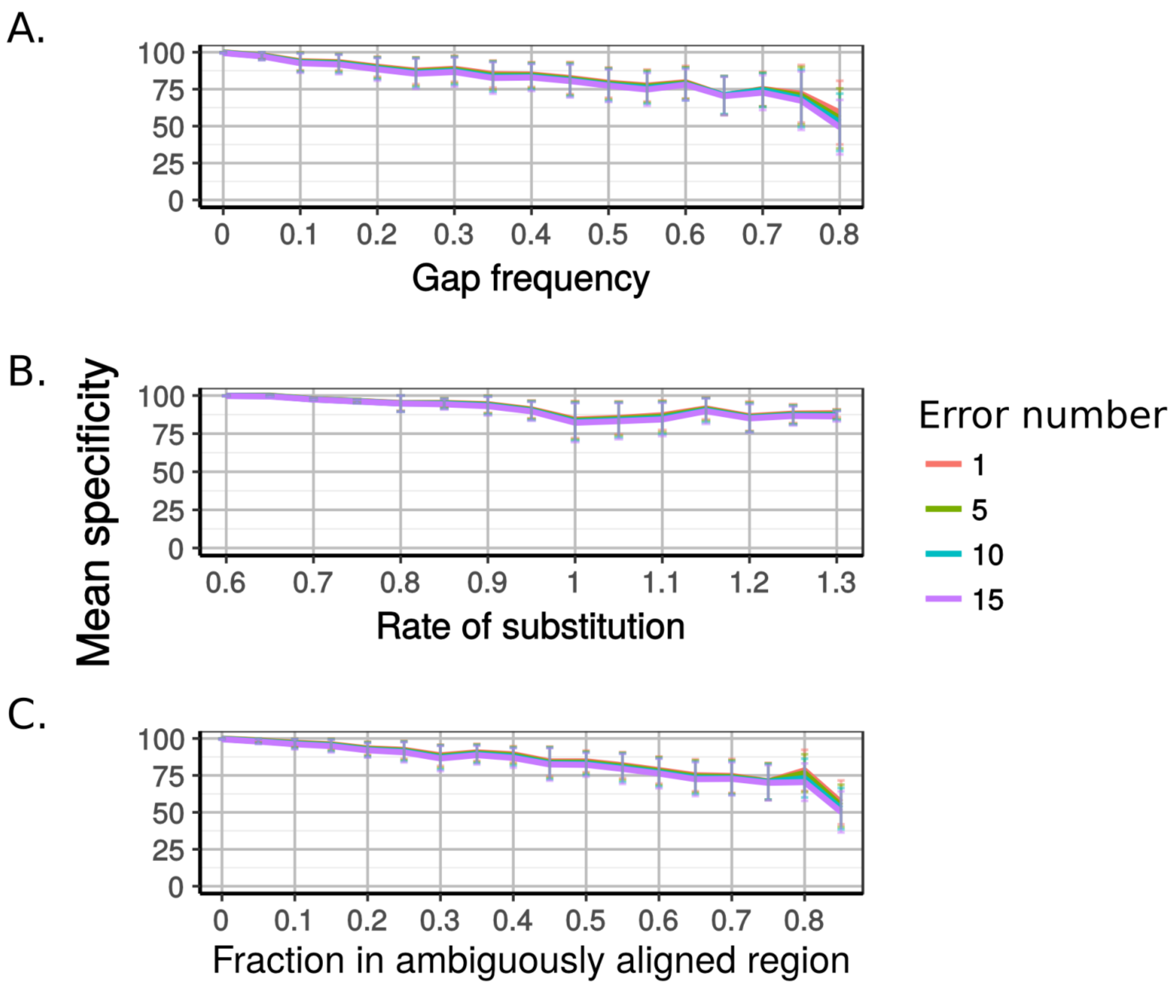

Suppl. Figure 7

Impact of the conservation context of introduced primary sequence errors on specificity of HmmCleaner used with the default scoring matrix for different numbers of errors. A. Specificity relative to the mean gap frequency in the MSA. B. Specificity relative to the mean rate of substitution in the MSA. C. Specificity relative to fraction of the MSA defined as AAR by BMGE (loose settings).
